# Carbon black supported Ag nanoparticles in zero-gap CO_2_ electrolysis to CO enabling high mass activity†

**DOI:** 10.1039/d3ra03424k

**Published:** 2023-06-21

**Authors:** Khaled Seteiz, Josephine N. Häberlein, Philipp A. Heizmann, Joey Disch, Severin Vierrath

**Affiliations:** a Electrochemical Energy Systems, IMTEK – Department of Microsystems Engineering, University of Freiburg Georges-Koehler-Allee 103 79110 Freiburg Germany severin.vierrath@imtek.uni-freiburg.de; b University of Freiburg, Institute and FIT – Freiburg Center for Interactive Materials and Bioinspired Technologies Georges-Köhler-Allee 105 79110 Freiburg Germany; c Hahn-Schickard Georges-Koehler-Allee 103 79110 Freiburg Germany

## Abstract

In this study Ag nanoparticles supported on carbon black (Ag/C) were studied as catalysts for the electrochemical reduction of CO_2_ to CO. The nanoparticles were synthesized on three carbon supports, namely Super P, Vulcan and Ketjenblack with surface areas from 50 to 800 m^2^ g^−1^ using cysteamine as a linker as proposed by Kim *et al., J. Am. Chem. Soc., 2015, **137**, 13844.* Gas diffusion electrodes were fabricated with all three Ag/Cs and then characterized in a zero-gap electrolyzer. All three supported catalysts achieve high voltage efficiencies, mass activities, and faradaic efficiencies above 80% up to 200 mA cm^−2^ with Ag loadings of ∼0.07 mg cm^−2^. Using an IrO_2_ anode, a partial CO current density of 196 mA cm^−2^ at 2.95 V and a mass activity of 3920 mA mg^−1^ at a cell voltage of 3.2 V was achieved. When changing the electrolyte from 0.1 M KOH to 0.1 M CsOH, it is possible to achieve 90% FE_CO_ at 300 mA cm^−2^. This results in a mass activity up to 5400 mA mg^−1^. Moreover, long-term tests at 300 mA cm^−2^ with 0.1 M CsOH resulted in FE_CO_ remaining above 80% over 11 h. The electrochemical performance did not show a dependence on the carbon support, indicating that mass transport is limiting the cathode, rather than catalyst kinetics. It is worth noting that this may only apply to electrodes with PTFE binders as used in this study, and electrodes with ionomer binders may show a dependence on the catalyst support.

## Abstract 2

In this study Ag nanoparticles supported on carbon black (Ag/C) were studied as catalysts for the electrochemical reduction of CO_2_ to CO.

## Graphical abstract

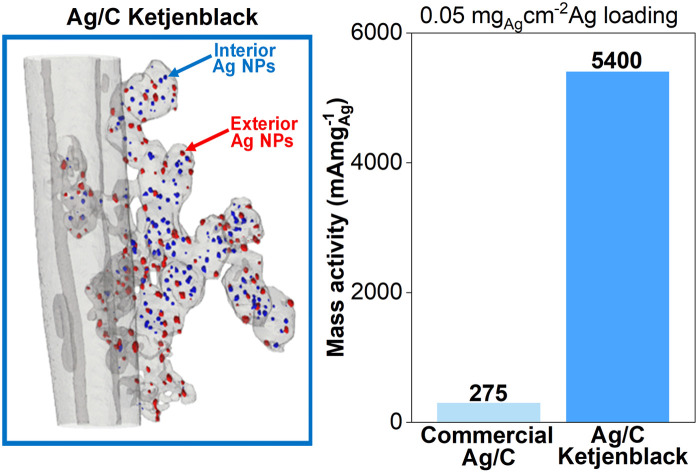

## 1. Introduction

To achieve global net-zero CO_2_ emission targets, we must defossilize all sectors that rely on carbon energy and fossil feedstock.^1^ Electrochemical reduction of CO_2_ is a very promising approach to defossilize the chemical industry and create a closed carbon loop.^2,3^ This technology enables the production of chemical feedstocks using captured CO_2_, water, and renewable energy. A wide range of cell designs and target products with carbon skeletons C_*x*_, usually ranging from *x* = 1–3 (*e.g.*, CO, formic acid, ethanol, *n*-propyl alcohol, and ethylene) are the subject of current research.^4–7^ The simplest product of electrochemical CO_2_ reduction is CO, which is a widely used feedstock for a wide range of industries.^8^ Hence, this work focuses on the production of CO *via* electrochemical CO_2_ reduction in a zero-gap electrolyzer cell. In this setup, the CO_2_ reduction catalyst is deposited onto a gas diffusion medium to form the so-called gas diffusion electrode (GDE). During operation the GDE is fed with humidified CO_2_, which is being reduced at the catalyst in the presence of water, forming CO and hydroxide anions (OH^−^).

Metal-based catalysts such as copper (Cu), gold (Au), silver (Ag), and zinc (Zn) are most commonly used to facilitate this reduction reaction. Besides catalytic activity also the selectivity plays a key role, especially towards the strongly competing hydrogen evolution reaction. Among these metal-based catalysts, Au has the highest selectivity for the production of CO, followed by Ag, Cu, and Zn.^9^ This is because Au and Ag have the optimal binding energy for the intermediate CO species, *i.e.* the intermediate CO species are strongly bound to the catalyst surface, preventing them from undergoing further reduction to other products.^10^

According to a study by Lim *et al.*, density functional theory (DFT) calculations suggest that the required overpotential for the electroreduction of CO_2_ to CO (CO_2_RR) can be significantly reduced (∼0.5 V) by doping Ag-based catalysts with sulfur (S) or arsenic (As).^11^ They proposed that the enhanced performance of the Ag–S or Ag–As catalysts is due to the introduction of covalent bonds on the Ag surface. The theoretical conjecture proposed by Lim *et al.* was subsequently confirmed by experimental work conducted by Kim *et al.* In their study, the researchers synthesized S doped Ag/C catalysts with varying Ag nanoparticle sizes using a simple one-pot synthesis with cysteamine as an anchoring agent.^12^ This approach has the advantage that it allows for the direct growth of immobilized Ag nanoparticles on a carbon support. The use of an anchoring agent, such as cysteamine, enables the formation of nucleation sites that promote the growth of monodispersed and homogeneous Ag nanoparticles, maximizing the surface area of the catalyst. Additionally, the anchoring agent is thought to change the electronic structure of the active sites on the Ag nanoparticles, as indicated by X-ray photoelectron spectroscopy and calculated by DFT. This is believed to result in more favorable binding energies between the intermediate CO species and the Ag nanoparticles, leading to a reduced overpotential for the reaction.^12^ They found that Ag nanoparticles with an average diameter of about 5 nm exhibit the highest CO_2_ reduction reaction activity with faradaic efficiencies up to 84.4% at −0.75 V (*vs.* RHE) in H-cells.

In addition to the work by Kim *et al.*,^12^ other researchers have also studied the effect of nanoparticle size on the activity and selectivity of Ag-based catalysts for CO2RR. For example, Deng *et al.* demonstrated that the CO_2_RR activity of Ag nanoparticles is mainly dominated by Ag(100) sites, with sub-5 nm Ag nanoparticles supported on highly oriented pyrolytic graphite being the optimal size for these catalysts.^13^ Similarly, Liu *et al.* synthesized Ag nanocubes that are fully enclosed by energetically favorable Ag(100) facets, which have a lower d-band center (−3.118 eV) than Ag(111) and edge sites (−3.646 eV and −3.586 eV, respectively).^14^ These studies highlight the importance of the size and surface structure of Ag nanoparticles for the performance of Ag-based catalysts in CO_2_RR. These three studies focused on testing the Ag nanoparticles in H-cells. Electrolysis carried out in H-type cells are susceptible to reach mass-transport limitations resulting in low current densities below 100 mA cm^−2^.^15^ Moreover, the dissolved CO_2_ reacts with OH^−^ to form carbonates in basic electrolytes.^16,17^ The consequence of that is the loss of CO_2_ decreasing CO_2_RR activity due to change of the pH value towards a more acidic environment, which affects the catalyst.^18,19^ Thus, H-type cells are not suitable for use in industrial conditions. In state-of-the-art CO_2_ electrolysis to CO non-supported Ag NPs are commonly used.^20^ However, supported catalysts can be more cost-effective compared to unsupported catalysts because the support material reduces the amount of catalytic material needed. Furthermore, the carbon support introduces an additional degree of freedom in the design, *e.g.* to ensure that reaction sites are well dispersed.

In this work, the synthesis of S-doped Ag/C catalysts developed by Kim *et al.*^12^ was adapted and applied in a zero-gap cell with a gas-fed cathode to show the potential in an industry relevant setup. This cell design enables high mass activities with low Ag loadings. Furthermore, an optimized synthesis was applied to various carbon supports in order to investigate the influence of the carbon surface morphology and Ag nanoparticle position on CO_2_RR activity.

## 2. Experimental

### 2.1 Materials

All reagents and solvents used in this study were purchased from commercial sources. Unless otherwise noted, these chemicals were used without further pretreatment or purification. For the Ag/C synthesis, silver nitrate (AgNO_3_, Sigma-Aldrich, 99.0%) was used as Ag precursor and cysteamine (Sigma-Aldrich) as anchoring agent. Carbon black Super P® conductive (Alfa Aesar, 99+%), carbon black Vulcan (Fuel cell store, Vulcan XC-72-R) and carbon black Ketjen (Nouryon, Ketjenblack EC-300J) were used as carbon support materials. Ethylene glycol (EG, Thermo Fisher, 99%), isopropyl alcohol (IPA, Carl Roth, >99.5%), methanol (MeOH, Carl Roth >99.5%) were used as solvents. Besides the synthesized Ag/C, commercial Ag/C (FC catalysts, 40% and 80% Ag on Vulcan) were used as cathode catalysts. Nickel iron oxide nanopowder (NiFe_2_O_4_, US Research Nanomaterials Inc, 99.99%) and iridium oxide nanopoweder (IrO_2_, Alfa Aesar, Premion 99.99%) were used as anode catalyst, respectively. Polytetrafluoroethylene (PTFE, Sigma-Aldrich, 60 wt% dispersion in H_2_O) was used as cathode catalyst layer binder, Aemion^+^ (Ionomer Innovations Inc., Aemion™ AP2-HNN8-00-X) as ionomer and zirconium oxide balls (ZrO_2_, Retsch, 5 mm) for grinding anode catalysts. Gas diffusion layer (GDL, Freudenberg carbon paper H23C6), Ni Felt (Bekaert, 200 μm), anion exchange membrane (AEM, Ionomer Innovations Inc., Aemion™ AF2-HNN8-50-X) and KOH (Sigma-Aldrich, pellets, > 85%) were used for cell testing. Lead(ii) acetate (Pb(ac)_2_, Sigma-Aldrich, ≥99.99%), sodium perchlorate (NaClO_4_, Sigma-Aldrich, 98%), perchloric acid (HClO_4_, Sigma-Aldrich, 70%) and sulfuric acid (H_2_SO_4_, Sigma-Aldrich, 98%) were used for surface area characterization. Cesium hydroxide (CsOH, Sigma-Aldrich, 99.95%) was used for long-term tests.

### 2.2 Ag/C synthesis

The synthesis of Ag/C was adapted and scaled up to different carbon supports as described by Kim *et al.*^12^ A simplified illustration of the synthesis can be seen in ESI Fig. S1.† First, carbon support (200 mg) in ethylene glycol (100 mL) was ultrasonicated (Bandelin Sonorex RK) for 30 min. Cysteamine (10 mg) was added to the carbon support solution and ultrasonicated for another 30 min. Then the mixture was added to a preheated (50 °C) solution of silver nitrate (200 mg) in ethylene glycol (100 mL), kept for 10 min at 50 °C before being heating up to 180 °C under reflux. The heating at 180 °C was maintained for 60 min. After cooling below 30 °C, the solution was washed with isopropyl alcohol, filtered and dried overnight in an oven at 80 °C. The final Ag/C catalysts were obtained as a black powder and yielded a mass between 200–300 mg, respectively.

### 2.3 Electrode preparation

Cathode GDEs were prepared by sonicating 100 mg of the respective Ag/C catalyst with 0.67 g of a 5 wt% PTFE dispersion (in H_2_O) in 7 mL H_2_O and 7 mL isopropyl alcohol for 30 minutes. The ink was then spray-coated onto the GDL with an ultrasonic spray coater (SNR 300, Sonocell). The hotplate below the GDEs was heated to 40 °C to accelerate solvent evaporation during ultrasonic spray-coating. Low Ag loadings with 0.05 mg_Ag_ cm^−2^ and 0.07 mg_Ag_ cm^−2^ for cathode Ag/C GDE's were obtained (Fig. S2†).

The anode ink was obtained by dispersing 600 mg NiFe_2_O_4_ or IrO_2_ in 1.8 mL H_2_O and 1.8 g of a 2.5 wt% Aemion^+^-solution (in MeOH/H_2_O 10/1 w/w). In addition, ZrO_2_ grinding balls were added. After 2 days on a roll mixer (IKA, Roller 10) the ink was casted with a bar coater (Mayer rod, 150 μm wet film thickness) onto the AEM to obtain the half catalyst coated membrane (HCCM) (Fig. S3†). 200 μm thick Ni-felts were used as anode electrode and cleaned by sonication in acetone, isopropyl alcohol and DI-H_2_O, consecutively.

### 2.4 Material characterization

#### Raman spectroscopy

Raman spectra of all samples were obtained with a WITec alpha 300 confocal Raman microscope equipped with a 532 nm laser excitation source. The shown spectra of the samples were produced at a power of 2 mW by averaging 5 single spectra, with each single spectra being integrated for 0.5 s and accumulated 100 times. For calculating the ratio of the D band and G band intensities (*I*_D_/*I*_G_) Lorentz fit was applied using WITec project. The error in the *I*_D_/*I*_G_ ratio was calculated using error propagation with the values obtained from the Lorentz fit.

#### Scanning electron microscopy (SEM)

Top view SEM micrographs of all samples were acquired with a FEG-SEM Amber X (Tescan GmbH) equipped with a secondary electron detector (Everhart–Thornley type). The samples were mounted on standard SEM Stubs (Science Services GmbH) with conductive double-sided adhesive carbon tabs. Micrographs were taken at a working distance of ∼6 mm, an acceleration voltage of 2 kV and a beam current of 100 pA.

#### Transmission scanning electron microscopy (S/TEM) and energy dispersive X-ray (EDX) mapping

A Talos F200X (S)TEM (ThermoFisher, high-brightness X-FEG emitter) equipped with a Ceta 16 Megapixel CMOS camera was used to record micrographs of the samples at 200 kV acceleration voltage. The samples were prepared by dispersing ∼100 μg catalyst powder in isopropyl alcohol, followed by brief sonication (Bandelin Sonorex super RK 100 H). Copper-based TEM grids (lacey carbon film, 3–4 nm nominal thickness, 200 quadratic mesh, ScienceServices GmbH) were loaded by dipping the grids into the solution and then dried in air. A model 2020 tomography holder (Fischione Instruments) was used for both acquisitions of 2D TEM micrographs and bright-field (BF)/high-angle annular dark-field (HAADF) tilt series image pairs (1024 × 1024 pixels, ± 72–75°, 2° tilt increment) in the STEM imaging mode. EDX mappings were acquired with a field of view of 300 nm. Elemental distributions were obtained with 100 scans and 15 μs dwell time per scan.

##### 2D analysis

The nucleation and growth behavior of the Ag nanoparticles may vary slightly depending on the carbon primary particle due to synthetically based chemical and structural inhomogeneities in the carbon support. For this reason, 2000 nanoparticles on at least 5 different Ag/C particles were considered to allow sufficient quantitative comparisons between all samples. All individual Ag nanoparticles were measured *via* ImageJ 1.53c.^21^

##### 3D analysis

The acquired tilt series image pairs were aligned by cross-correlation using Inspect3D. By tracking individual Ag nanoparticles on the sample, this cross-correlation was further refined (see ESI videos 00 and 01† for an exemplary comparison before and after image alignment). The micrographs were then binned by *a* factor of 2, followed by reconstruction of the tomogram by using an OSEM algorithm with 20 iterations.^22^ This provided sufficient tomogram quality to distinguish Ag nanoparticles sitting on the exterior surface of the carbon support from nanoparticles inside the support. The tomograms were segmented in ImageJ iteratively using the Trainable Weka Segmentation plugin and visualized with the open source tomography platform TomViz.^23,24^ Due to methodological (*e.g.*, missing-wedge artifacts due to limited tilt range) and experimentally introduced artifacts (*e.g.*, possible slight intraparticle movements throughout the acquisition), only the position of the nanoparticles but no other morphological aspects were analyzed, also because the direct evaluation of particle diameters from the raw 2D TEM micrographs in our opinion is more precise due to the not required intermediate processing steps and statistically more robust ascribable to the increased number of considered Ag/C parent particles.

#### Thermogravimetric analysis

A NETZSCH STA 409C/CD (Netzsch-Geraetebau GmbH) was used for thermogravimetric analysis (TGA). TGA was carried out under oxygen atmosphere with a heating rate of 10 °C min^−1^ and flow rate of 100 mL min^−1^. TGA experiments were performed on Ag/C catalysts up to 930 °C under an oxygen atmosphere to investigate the decomposition of the carbon black support and determine the Ag loading (Fig. S4†). A significant weight loss above 400 °C is due to the decomposition of the carbon black. At around 700 °C, the carbon black particles are completely burned. From 700 to 930 °C, the mass weight remains constant. At 930 °C, the weights were recorded as they correspond to the residual Ag, as the melting point of Ag is around 961 °C.^25^

#### X-ray fluorescence

A Bruker M4 TORNADO was used for micro X-ray fluorescence (XRF) measurements to determine the loading of the Ag catalyst on the GDE. The loading was extracted using the Bruker XMethod software. Under vacuum, an elemental mapping of the respective prepared electrode (approximately 2 × 4.5 cm area) was obtained with a source current of 200 μA, a beam incidence angle of 50° and a scan rate of 30 ms per point.

#### Contact angle measurements

Contact angle measurements of electrodes were obtained using a Dataphysics OCA 15 plus. The samples were 2 cm^2^ in size, and 5 μL of water droplet was applied to the surface.

### 2.5 Electrochemical measurements

#### Cell measurements

Before assembly (Fig. S5†), the AEM coated with the anode catalyst layer was pretreated in 1 M KOH solution for 24 h. 95 sccm of CO_2_ was humidified at (23 ± 3) °C fed to the cathode side and 0.5 L of a 0.1 M KOH solution was supplied to the anode side at a flow rate of 20 mL min^−1^ with a peristaltic pump (Reglo ICC 7800-58, Ismatec). Electrochemical experiments were conducted using a Bio-Logic Galvano-/Potentiostat VSP-300. An Agilent micro gas chromatograph (GC) 990 equipped with a two channels (Molsieve 5 Å column with argon as carrier gas and a PoraPLOT Q column with helium as carrier gas) was used for analyzing the composition of the output gas. GC measurements were conducted after 10 minutes of each current step. The GC was connected online to the outline of cathode after a water trap, a gas drying unit (gasmet) and a mass flow meter (Bronkhorst). The cell performance was evaluated by applying different current densities from 12.5 mA cm^−2^ to 300 mA cm^−2^. Each current was applied for 10 minutes and at the end of each current step, a galvanostatic electrochemical impedance spectroscopy measurement (1–500 kHz and 5% amplitude) was conducted to determine the high frequency resistance. For long-term operation, a constant current of 100 mA cm^−2^ with 0.1 M KOH and a high current density of 300 mA cm^−2^ with 0.1 M CsOH were applied. Every 10 minutes, a galvanostatic electrochemical impedance spectroscopy measurement and GC measurements were (similar to current density steps) conducted.

#### Electrochemical calculations

The *iR*-corrected cell potential (*U*_*iR* corrected_) was obtained as follows:*U*_*iR* corrected_ = *U*_cell_ − *i*·HFR_*i*_.Herein*, U*_cell_ is the measured cell potential, *i* denotes current density, and HFR_*i*_ denotes the high-frequency resistance at a given current density. The respective high-frequency resistance was determined by obtaining the *x*-intercept of the Nyquist plot of the electrochemical impedance spectroscopy (EIS) measurements at each current density. The following equation was employed to evaluate the faradaic efficiency FE_*x*_ (with *x* = H_2_ or CO_2_) of the products:
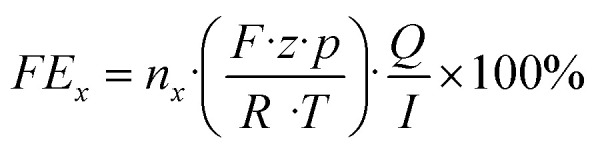
Herein, *n*_*x*_ denotes the measured mole fraction of the product of interest as quantified by GC, *F* is the Faraday constant (96 485 C mol^−1^), *R* the ideal gas constant (8.314 J K^−1^ mol^−1^), *Q* the volumetric flow rate, *T* the temperature, *z* the charge transfer number, *p* the pressure and *I* the total current. By multiplying the product-specific Faraday efficiency with the total current density, the product-specific partial current density *i*_*x*_ can be obtained.

#### Electrochemical active surface area

To determine the electrochemical active surface area (ECSA) lead underpotential deposition (Pb UPD) in 1 mM Pb(acetate)_2_ + 1 mM HClO_4_ + 0.5 M NaClO_4_ solution was performed. A three electrode setup was used consisting of a saturated calomel electrode (SCE) as reference electrode, a Pt mesh as counter electrode and the respective GDE as working electrode. The CV was measured between 0 V and −0.75 V using a scan rate of 5 mV s^−1^. The desorption peak of the Pb UPD correlates with the ECSA.^26^

#### Electrochemical double-layer capacitances

The electrochemical double-layer capacitances (EDLC) of all samples were determined by cyclic voltammetry (CV) in a potential range of 0.2 to 0.3 V *vs.* SCE in 1 M H_2_SO_4_. The charging current was measured at 0.26 V six times at different scan rates (*v =* 5, 25, 50, 100, 150 and 200 mV s^−1^) and plotted against the scan rates. As the capacitive current is proportional to the rate of change of the voltage d*V*/d*t*, EDLC was then extracted from the slope.^27^

## 3. Results & discussion

The influence of carbon support was investigated by applying the synthesis method described in the experimental section to three different common and widely used carbon supports: Ketjenblack with a high surface area of approximately ∼800 m^2^ g^−1^, Vulcan with a medium surface area of ∼200–250 m^2^ g^−1^ and carbon black Super P with a low surface area of ∼50 m^2^ g^−1^.^28–31^ TEM micrographs show that the carbon supports are composed of primary particles with sizes in the range of 20–60 nm, which then form larger aggregates with sizes of approximately 250–1000 nm (Fig. S6–S8†). The micrographs further indicate that the primary particles of the carbon supports consist of a shell of graphitic carbon and a core of amorphous carbon. The greatly increased surface area of the Ketjenblack sample presumably is due to the degree of hollowing of the carbon support rather than the primary particle sizes. In literature, pore volumes of ∼2 m^3^ g^−1^ are reported for Ketjenblack, compared to ∼0.3 m^3^ g^−1^ for Super P (see the ESI† for a detailed characterization of the carbon supports).^32^

**Fig. 1:**
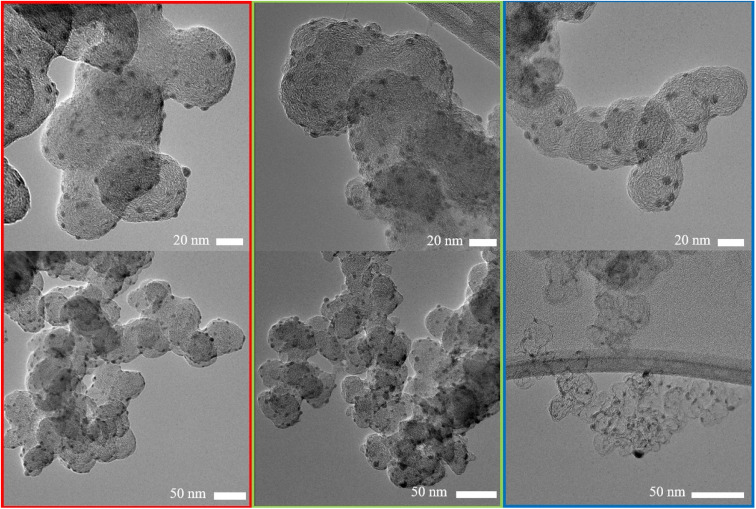
Representative TEM micrographs of the synthesized Ag/C samples. Red: Ag/C_Super P_, green: Ag/C_Vulcan_, blue: Ag/C_Ketjen_.

Representative TEM micrographs of all synthesized Ag/Cs can be seen in Fig. 1. No significant change in carbon support morphology due to nanoparticle synthesis was observed. For the three carbon substrates shown, spherical Ag nanoparticles are homogeneously distributed over the entire carbon support surface. The average nanoparticle diameter was found to be inversely correlated to the surface area: Ag/C_Super P_ (5.3 nm ± 1.5 nm) > Ag/C_Vulcan_ (4.7 nm ± 1.5 nm) > Ag/C_Ketjen_ (4.4 nm ± 1.3 nm) (Fig. S14–S16†). This is potentially a consequence of the sharp increase in surface area from Super P to Ketjenblack with concomitant increase in the number of Ag crystallization nuclei and the decrease of probability of agglomeration of individual nanoparticles. Although a strong dependence of nanoparticle size and CO_2_ activity has been reported, the large overlap of the histograms (Fig. S17†) and the marginal difference of the average particle diameter from 4.4 nm to 5.3 nm allows comparing the three samples.^13^ The catalyst loadings were 31 wt% for Ag/C_Super P_ < 33 wt% for Ag/C_Vulcan_ < 37 wt% for Ag/C_Ketjen_ as determined by TGA (Fig. S4†). Again this trend could be explained by the increasing surface area and thus nuclei and deposition using the same synthesis parameters for all three carbons.

As reported for fuel cells, the position of the catalyst on the carbon support can have a significant impact on the performance. In general, nanoparticle catalysts on the surface of the carbon support have better access to reactants leading to higher reaction rates. However, an adverse effect can be observed for fuel cells, when catalyst particles on the carbon surface are rendered inactive by adsorbing functional groups of the ion-conductive binder.^33^ As a consequence, electrodes in fuel cells are optimized depending on the desired activity and reactant accessibility by choosing a carbon support with the right surface area. Typically, low surface area carbons lead to particles being dispersed on the outer surface of the carbon, while higher surface carbon features mesopores that contain a large fraction of the catalyst particles.^34^

**Fig. 2:**
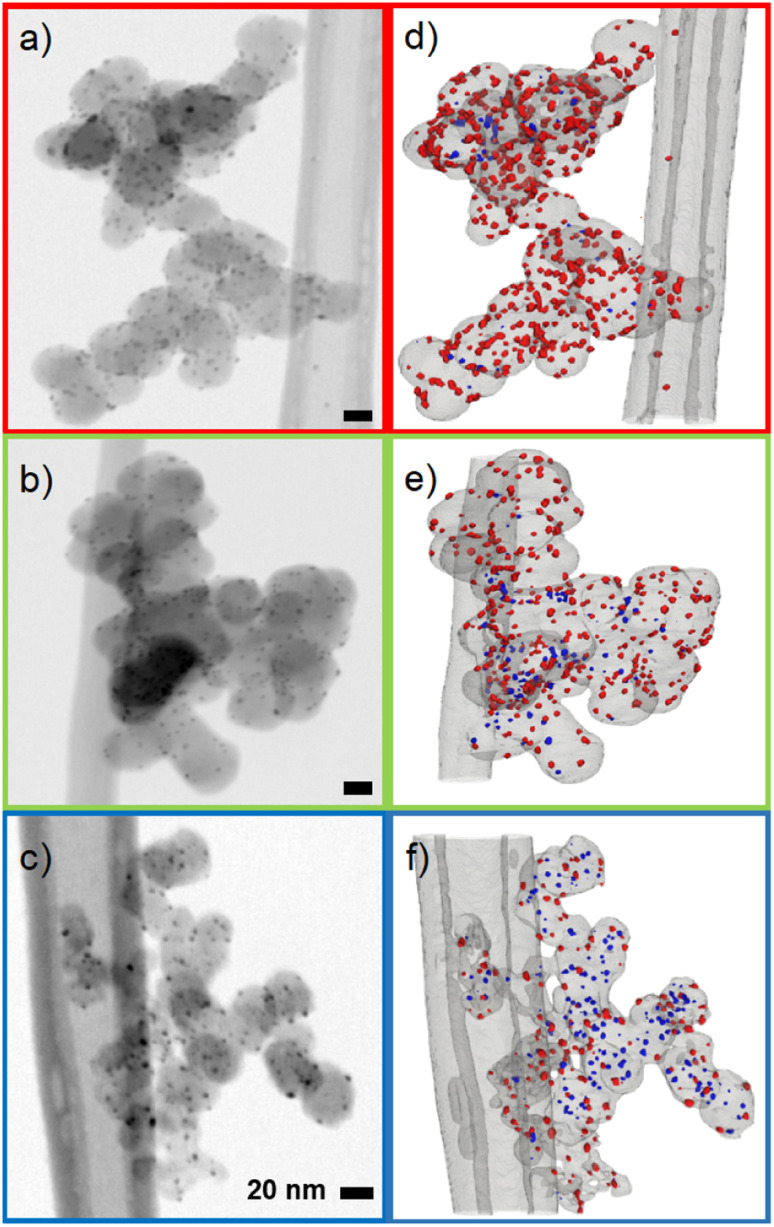
Electron tomography of Ag synthesized on three carbon blacks: Ag on Super P (red), Ag on Vulcan (green), Ag on Ketjenblack (blue). (a–c): Representative BF STEM micrographs of the Ag/C samples. (d–f): Segmented volumes showing interior (blue) and exterior Ag nanoparticles (red). Note that the segmented volumes contain depth and thus the 2D *vs.* 3D projections do not match exactly in this representation.

**Fig. 3:**
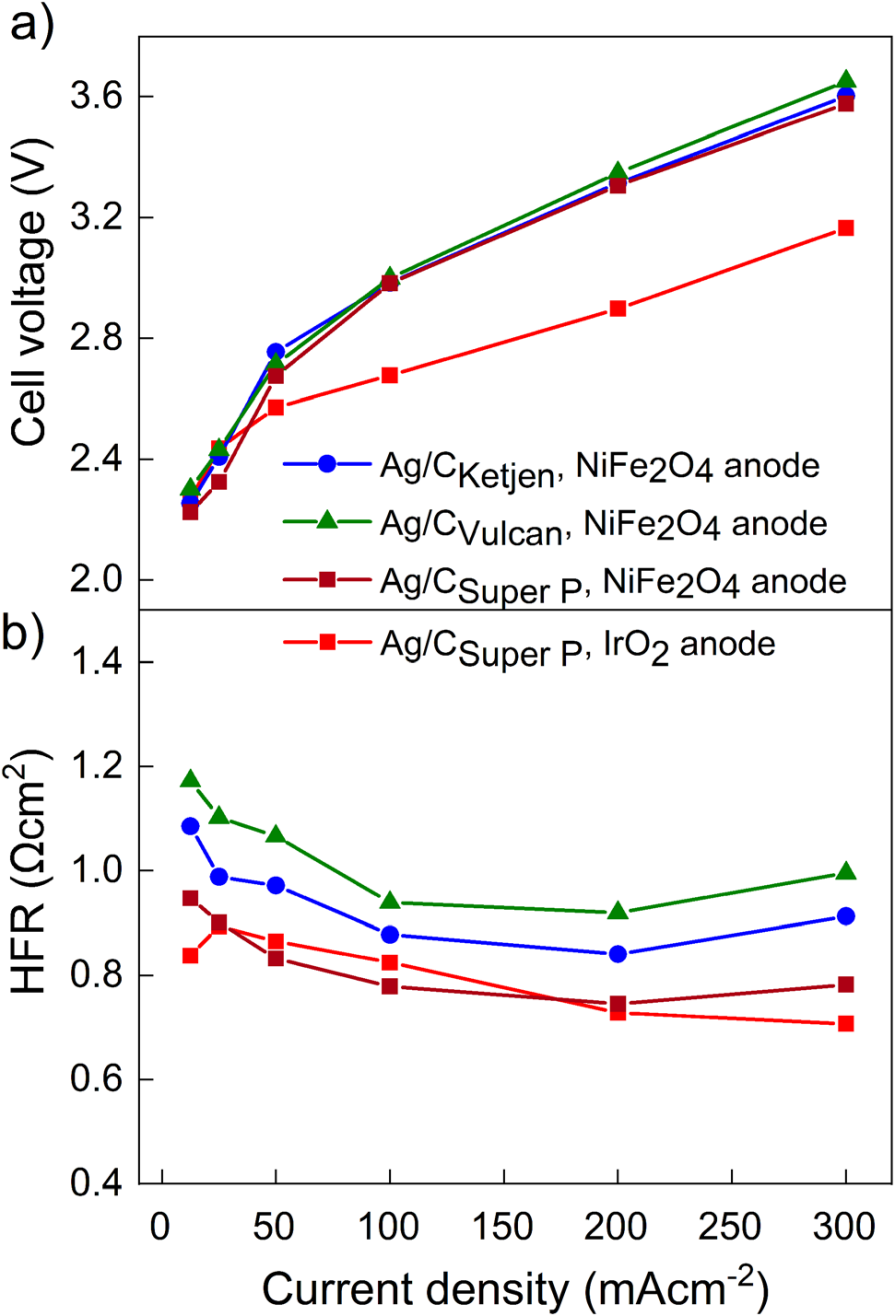
(a) Cell voltages and (b) high frequency resistances of Ag/C_Ketjen_, Ag/C_Vulcan_ and Ag/C_Super P_ catalysts in 0.1 M KOH at ambient temperature. 25% PTFE is used as cathode catalyst binder and NiFe_2_O_4_ as anode catalyst. Only for second Super P sample IrO_2_ was used as anode catalyst.

To determine the position 3D STEM tilt series were recorded (Fig. 2a–c) and 3D volumes were reconstructed (Fig. 2d–f, ESI videos 02, 03, 04 and Fig. S18†). As can be seen in Fig. 2, the proportion of internal particles increases from ∼10% for Super P to 20% for Vulcan and ∼65% for Ketjenblack. This finding is consistent with studies on similar Pt/C catalysts, and is likely due to the higher porosity of the Ketjenblack carbon support. For example, Padgett *et al.* reported an interior fraction of around 0.2 for 10 wt% Pt/Vulcan, and Sneed *et al.* reported an interior fraction of around 0.7 for 20 wt% Pt/Ketjenblack.^29,35^ (see the ESI† for a detailed discussion of the TEM data).

It is difficult to determine the precise fraction of nanoparticles that are inside or outside the carbon pores based on 2D TEM micrographs. However, some nanoparticles can be assigned to the outside of the carbon support if they are clearly visible outside the projection of the carbon primary particle, as shown by Harzer *et al.*^36^ In turn, if a nanoparticle is totally encompassed by the carbon particle's projection, it cannot be distinguished whether it is on the surface or in a carbon pore. From this derivation, a qualitative decrease in exterior particles in the order Super P > Vulcan > Ketjenblack was observed. To better estimate the fraction of interior and exterior nanoparticles, 3D STEM tilt series were recorded and 3D volumes were reconstructed (see ESI videos 02, 03, 04 and Fig. S18†). We make no claim to perfect reconstructions, as tilt series based electron tomography has method-based problems (*e.g.*, the missing wedge problem introducing elongation and ghost tail artifacts) and the samples themselves are difficult to investigate (*e.g.*, limited radiation dose due to rapid degradation of the carbon species).^37^ For an in-depth analysis of the issues involved in STEM tomography of metal/carbon based catalysts, the reader is referred to the highly recommended paper by Padgett *et al.*^34^ Nevertheless, the position of an individual nanoparticle can already be estimated much more accurately from the raw tilt series than with single projection 2D micrographs (Fig. S19 and ESI video 05†), and the reconstructed volumes obtained allow sufficient discrimination between interior and exterior nanoparticles.

Raman spectroscopy was used to identify the Ag–S bond formation in the Ag/C catalysts and potential changes in the graphitic structure of the various carbon supports. Sadovnikov *et al.* investigated nanostructured Ag sulfide using Raman spectroscopy and found a series of vibrations in the range of 90–260 cm^−1^ caused by Ag–S bonds.^38^ The Raman spectra of our synthesized Ag/C catalysts also show Raman bands at 90–220 cm^−1^, which indicates the formation of Ag–S bonds (Fig. S20†). This is further confirmed by the absence of near field Raman bands in the case of the various carbon supports and the commercial Ag/C_Vulcan_ catalysts. In addition, the Ketjenblack samples contain more structural disorder and defects, as indicated by the highest D to G band ratios (Fig. S21†). The following trend can be observed when comparing carbon blacks: Ketjenblack has the highest defect concentration (*I*_D_/*I*_G_ ratio of 1.18), followed by Vulcan (ratio of 1.08), and then Super P (ratio of 0.85). This is due in part to Ketjenblack having a higher BET surface area, which can result in a larger number of surface defects. This increase in surface defects can contribute to an increase in the intensity of the D band in the Raman spectra. The *I*_D_/*I*_G_ ratios for Ketjenblack, Vulcan and Super P are consistent with the ratios reported in the literature.^39,40^ It's interesting to note that Ag supported catalysts demonstrate higher D to G band ratios compared to the respective carbon black. The higher ratio can be attributed to the presence of Ag NPs on the surface of the carbon black, inducing more structural disorder, which in turn increases the intensity of the D band in Raman spectra. Note that a low laser power of 2 mW was used for these measurements due to the fragile nature of the carbon supports.

The ECSA was evaluated using Pb UPD (Fig. S22†). The pristine GDL only showed bulk Pb deposition starting at −0.6 V *vs.* SCE and more negative potentials, indicating no contribution to the active surface area. However, Pb UPD and Pb monolayer desorption on Ag could be obtained with the Ag/C catalysts. The ECSA was estimated by the Pb-UPD desorption peak.^26^ The ECSA of the Ag/C catalysts showed a slight trend in the order Ag/C_Ketjen_ > Ag/C_Vulcan_ > Ag/C_Super P_, which can be explained with their increasing average particle size of 4.4 nm, 4.7 nm, and 5.3 nm (Fig. S14–S16†).

Additionally, EDLC measurements were conducted (Fig. S23†). CVs in the non-faradaic region from 0.2–0.3 V *vs.* SCE were recorded, and the charging current was plotted against the scan rate. The slope of the regression line represents the EDLC value. The EDLC of the pristine GDL was negligible due to its low wettability properties with a water contact angle of 144°, resulting in no significant formation of a double-layer (Fig. S24†). The EDLC values were calculated as 18.7 F g^−1^ for Ag/C_Super P_, 43.9 F g^−1^ for Ag/C_Vulcan_, and 103.6 F g^−1^ for Ag/C_Ketjen_. The increase in EDLC is the result of the increasing carbon surface area and increased wettability from Ag/C_Super P_ (water contact angle of 127°) to Ag/C_Ketjen_ (water contact angle of 103°, Fig. S24†). Tashima *et al.* showed that a highly porous Ketjenblack (BET = 1445 m^2^ g^−1^) had the highest EDLC value of 59.2 F g^−1^ compared to acetylene black with a small BET of 66 m^2^ g^−1^.^41^ Therefore, the significant change in EDLC for the Ag/C catalysts is mainly due to the carbon support and should not be considered a measure for active area of (supported) catalysts. In contrast, Pb-UPD selectively measures the surface of Ag.

The performance of the three carbon supports with Ag catalysts was assessed using a zero-gap electrolyzer. Ag/C_Ketjen_, Ag/C_Vulcan_, and Ag/C_Super P_ gas diffusion electrodes were assembled with an Aemion^+^ 50 μm membrane and a NiFe_2_O_4_ anode. A PTFE particle dispersion was used as a cathode catalyst binder to minimize the influence of interaction between binder and catalyst particles. The PTFE particles increase the hydrophobicity in the catalyst layer, potentially helping to mitigate electrode flooding.^42^Fig. 3a shows the cell voltage dependent on current density for cells containing Ag/C_Ketjen_, Ag/C_Vulcan_, and Ag/C_Super P_ with no significant trend. For instance, at 100 mA cm^−2^ cell voltages were between 2.95 V and 3 V for Ag/C catalysts, respectively. When changing the anode catalyst to IrO_2_ the cell voltage was reduced by 300 mV to 2.65 V with Ag/C_Super P_ as cathode catalyst due to the higher OER activity of IrO_2_ compared to NiFe_2_O_4_.^43,44^Fig. 3b shows the high-frequency resistance (HFR) with a trend in the order of Ag/C_Super P_ < Ag/C_Ketjen_ < Ag/C_Vulcan_, resulting in slightly lower cell voltages of Ag/C_Super P_ with 3.58 V compared to Ag/C_Vulcan_ with 3.65 V at high current density (300 mA cm^−2^). The cell voltage and resistance measurements were repeated showing negligible deviation with cell voltages being between 2.95 V at and 3 V at 100 mA cm^−2^ confirming reproducibility (Fig. S25†). Overall, the results showed that the carbon support does not have a significant effect on the performance of the Ag/C catalysts. Furthermore, we conducted cell tests using Super P as the carbon support for the cathode catalyst in combination with an IrO_2_ anode (Fig. S26†). The cell demonstrated a maximum *j*_CO_ of 0.84 mA cm^−2^ when a current density of 100 mA cm^−2^ was applied at 3.78 V. These results suggest that the carbon support does not exhibit any significant catalytic activity for the reduction of CO_2_ to CO. Therefore, the CO mass activity can be predominantly attributed to the presence of Ag on the Ag/C catalysts.

In AEM zero-gap CO_2_ electrolysis, cross-over of CO_2_ gas from the cathode to the anode is always present, altering the pH of the anolyte. This reduces the overall performance of the cell. Janáky *et al.* found that to conduct long-term studies, the anolyte must either be frequently replenished to maintain a high alkaline pH or an anode catalyst that can withstand OER at close to neutral pH must be used.^45^ However, in our study the pH change during the measurements was small due to the short measurement duration.

**Fig. 4:**
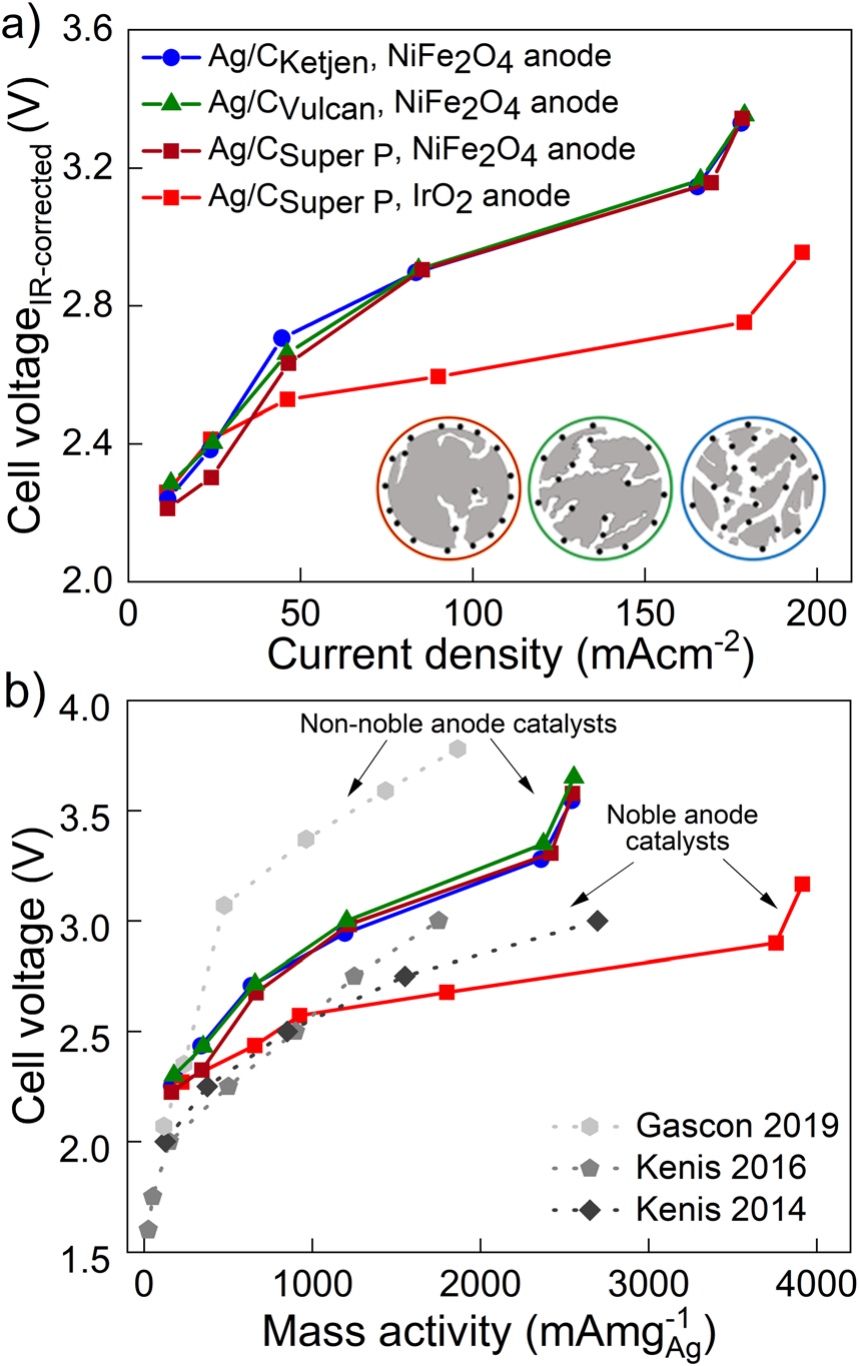
(a) *iR*-Corrected cell voltage over CO partial current density for Ag/C_Ketjen_, Ag/C_Vulcan_ and Ag/C_Super P_ catalysts in 0.1 M KOH at ambient temperature. (b) CO partial current density relative to Ag loading of selected supported Ag catalysts achieving high mass activities.^46–48^

Fig. 4a shows the *iR*-corrected cell voltage, *i.e.* without ohmic contributions from *e.g.* the membrane, over CO partial current density. Similar partial current densities *j*_CO_ of 178 mA cm^−2^ at 3.3 V were obtained for all Ag/C catalysts with NiFe_2_O_4_. The faradaic efficiency FE_CO_ for the Ag/C catalysts remained above 80% up to 200 mA cm^−2^, while it drops to 59% at 300 mA cm^−2^ (Fig. S27†). The cells reproduced using Ag/C catalysts maintained FE_CO_ above 80% up to 200 mA cm^−2^. Again these results indicate that the type of carbon support on the Ag/C catalyst does not have a significant effect on the electroreduction of CO_2_ to CO in our system. This is likely because the Ag/C catalysts have similar particle size distributions with average diameters of around 5 nm. It further indicates that the electrode binder (PTFE) does not poison or block the active catalyst particles. This is an indicator that the peak performance is not limited by kinetics from the catalyst but by other mass transport limitations that affect all supported catalysts similarly. Cell performance with IrO_2_ on the anode side, however, lead to a maximum *j*_CO_ of 196 mA cm^−2^ at 2.95 V with FE_CO_ remaining over 90% up to 200 mA cm^−2^. In contrast, with commercial Ag/C_Vulcan_ catalysts we were not able to produce current densities greater than 30 mA cm^−2^ (Fig. S28†). This poor performance is likely due to the large, unbound Ag nanoparticles with low surface-to-volume ratios (Fig. S29 and S30†).

The Ag loading on the cathode GDEs with the synthesized Ag/C catalysts was determined to be 0.07 mg_Ag_ cm^−2^ by XRF (Fig. S2†), except for the Ag/C_Super P_ GDE with IrO_2_ on the anode side (0.05 mg_Ag_ cm^−2^).

The performances of the various catalysts normalized by the mass of Ag are compared in Fig. 4b. The Ag/C catalysts presented here demonstrate high mass activities amongst reported literature for carbon supported Ag catalysts (see Table 1): 2543 mA mg^−1^ for Ag/C_Ketjen_, 2556 mA mg^−1^ for Ag/C_Vulcan_ and 2547 mA mg^−1^ for Ag/C_Super P_ with NiFe_2_O_4_ anodes. Gascon and coworkers achieved a high mass activity of 1864 mA mg^−1^ at 3.78 V with porous carbon supported Ag (Ag/MPL).^46^ This high cell voltage is comparable to our cell voltages (3.54–3.65 V) of the peak mass activity and probably characteristic for non-noble Ni anode catalysts. In contrast, Kenis and coworkers achieved remarkable mass activities up to 2696 mA mg^−1^ at 3 V with supported Ag catalysts and noble anode catalysts.^47–49^ For comparison, our Ag/C_Super P_ GDE with noble IrO_2_ catalyst exhibited an outstanding mass activity of 3920 mA cm^−2^ with 0.1 M KOH at 3.16 V surpassing the reported literature data on supported Ag and Au catalysts as summarized in Table 1.

**Table 1:** Comparisons of CO2RR to CO catalytic activity of supported Ag and Au catalysts in flow cells

Supported Ag/Au catalyst	Metal loading (mg cm^−2^)	Metal NP size (nm)	Anode catalyst	Electrolyte	*j*_CO, max_ (mA cm^−2^)	Cell voltage at *j*_CO, max_ (V)	Mass activity (mA mg^−1^)	Reference
Ag/C_Ketjen_	0.05	4.4 ± 1.3	IrO_2_	0.1 M CsOH	270	3.34	5400	This work
Ag/C_Super P_	0.05	5.3 ± 1.5	IrO_2_	0.1 M CsOH	261	3.38	5220	This work
Ag/C_Super P_	0.05	5.3 ± 1.5	IrO_2_	0.1 M KOH	196	3.16	3920	This work
Ag/C_Ketjen_	0.07	4.4 ± 1.3	NiFe_2_O_4_	0.1 M KOH	177.9	3.55	2543	This work
Ag/C_Vulcan_	0.07	4.7 ± 1.5	NiFe_2_O_4_	0.1 M KOH	178.9	3.65	2556	This work
Ag/C_Super P_	0.07	5.3 ± 1.5	NiFe_2_O_4_	0.1 M KOH	178.3	3.58	2547	This work
5 wt% Ag/TiO_2_	0.017	70	Pt	1 M KOH	45.6	3	2696	Kenis 2014 (ref. 47)
Ag/MPL-3C	0.20	25–30	Ni mesh	0.1 M KHCO_3_	385	3.8	1864	Gascon 2019 (ref. 46)
AgNP/MWCNT	0.20	—	IrO_2_	1 M KOH	350	3	1750	Kenis 2016 (ref. 48)
Au_25_(SR)_18_/CB	0.00096	1.4 ± 0.4	Pt	0.1 M KHCO_3_	1.59 ± 0.16	2.73	1656 ± 163	Jin 2015 (ref. 59)
Au_24_NHC/Carbon	0.066	—	IrO_2_	0.1 M KHCO_3_	90	3.2	1360	Crudden 2022 (ref. 60)
Au/C	0.4	11	IrO_2_	H_2_O	500	3	1250	Zhuang 2019 (ref. 61)
Au/CN	0.2	2–3	RuIrO_*x*_	2 M KOH	180	3	900	Wu 2022 (ref. 62)

**Fig. 5:**
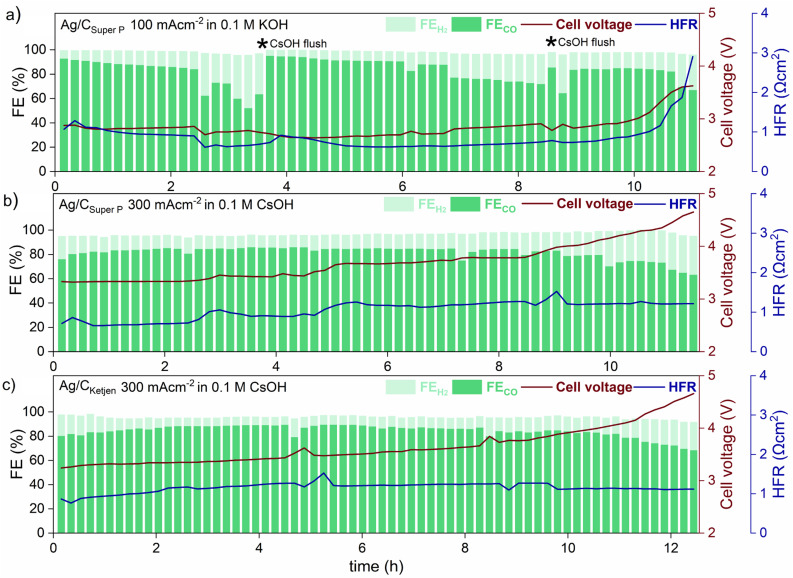
Long-term operation at ambient temperature of (a) Ag/C_Super P_ with a 0.05 mg_Ag_ cm^−2^ at 100 mA cm^−2^ in 0.1 M KOH, (b) Ag/C_Super P_ and (c) Ag/C_Ketjen_ with 0.05 mg_Ag_ cm^−2^ at 300 mA cm^−2^ in 0.1 M CsOH. A membrane coated with a IrO_2_ catalyst layer for the anode side was used for all Ag/C catalysts. The depicted * in (a) represents cathode flush by 50 μL 0.1 M CsOH to remove accumulated carbonates on the surface of the cathode.

Long-term tests with Ag/C_Super P_ and Ag/C_Ketjen_ were conducted as shown in Fig. 5. In the first long-term measurement with Ag/C_Super P_, 0.05 mg_Ag_ cm^−2^ in 0.1 M KOH at 100 mA cm^−2^ for 11 h (shown in Fig. 5a), the FE_CO_ remained above 80% for 2 hours, but then dropped to 60%. This decrease in FE_CO_ was likely due to the accumulation of potassium carbonates on the cathode side, which can lower the kinetics towards CO_2_ to CO reduction and result in reduced current densities.^45^ To address this issue, regular cathode flushs with 50 μL 0.1 M CsOH were used to remove any accumulated carbonates on the surface of the cathode, which successfully restored FE_CO_ to above 80%.^50^

The formation of salt precipitation can be better controlled by changing the cation of the anolyte: Cofell *et al.* observed an increase in the performance caused by lower carbonate salt precipitation could be obtained by changing the electrolyte from KOH to CsOH.^51^ This effect can be ascribed to the larger Cs^+^ cations having a smaller hydration shell compared to K^+^ resulting in being more concentrated on the electrode surface. This is believed to stabilize the CO_2_ reduction intermediate species, contributing to improved kinetics.^52,53^ Moreover, cesium hydrogen carbonates are more soluble in water than potassium carbonates (CsHCO_3_ with 13.16 molkg^−1^*vs.* KHCO_3_ with 3.64 molkg^−1^), indicating a reduced tendency for precipitation in Cs-based electrolytes.^51,54^ To make use of this, Ag/C_Super P_ and Ag/C_Ketjen_ with 0.05 mg_Ag_ cm^−2^ were tested in 0.1 M CsOH (Fig. 5b and c). As can be observed, switching the electrolyte from KOH to CsOH yields favorable long-term operation at 300 mA cm^−2^. The cell performance show that the FE_CO_ of the cell remained above 80% for a period of 9 h when using Ag/C_Super P_, and over 11 h when using Ag/C_Ketjen_. These findings were consistent with similar overall cell voltages and HFR values.

Both cells demonstrated gradual increase in cell voltage over time. This increase in cell voltage for Ag/C_Super P_ and Ag/C_Ketjen_ was found to be likely due to a change in pH value during electrolysis (Fig. S31†). It was observed that the pH values of all Ag/C catalysts undergo a shift from an initial range of ∼13–14 to a final range of ∼8–9. Erikson *et al.* reported a fast decrease in pH from 14 to 8 with AEMs, which resulted in a 1.2 V increase in cell voltage.^55^ The observed increase in cell voltage for Ag/C catalysts was also in a similar range, with Ag/C_Ketjen_ showing a 1.33 V increase (from 3.24 V to 4.57 V after 12.5 h) and Ag/C_Super P_ showing a 1.25 V increase (from 3.32 V to 4.57 V after 11.5 h). The pH value of the electrolyte plays a crucial role in OER kinetics, which impacts the overall cell voltage. After long-term tests, it was observed that Ni-felts degraded, since they are known to be highly unstable in a neutral medium causing the increase in cell voltage over time (Fig. S32†).^45^

After having clarified that the voltage increase does not stem from nanoparticle degradation, we conducted post-test TEM imaging of the Ag/C GDEs after long-term operation to show that the majority of the Ag NPs retain their size (Fig. S33†). Hence, in the presented measurements, no significant catalyst degradation was observed, as the performance degradation was superimposed by other degradation mechanisms.

This is in line with the fact that thiol-based linkers are widely used due to strong covalent bond with metal centers to prevent the degradation and aggregation of Ag NPs.^56,57^ As a result, the surface of the Ag NPs can be effectively passivated, which reduces the possibility of degradation and aggregation. Adding to this, citrate capped NPs can exhibit irreversible aggregation behavior due to the weak binding to the metal surface.^57,58^ These findings suggest thiol-based linkers as promising candidates for achieving long-term durability.

## 4. Conclusion

In this study, we synthesized Ag catalysts supported on three carbon blacks, Ketjenblack, Vulcan and Super P carbon, for electrochemical CO_2_ reduction to CO. The synthesized Ag nanoparticles were all evenly dispersed on the three carbon supports with a loading of ∼30 wt% Ag on carbon. The obtained average diameter of about 5 nm is believed to be the ideal particle size towards electrochemical CO_2_ reduction to CO. A slight trend for the surface area of the carbon supports was observed with increasing surface area leading to smaller Ag particles. As a consequence the electrochemical active surface area as determined by Pb under potential deposition is also higher for the catalyst with higher surface area carbons. TEM tomography revealed that the mesoporous structure of the carbon support has a major effect on the final position of the deposited particles, as known from carbon supported Pt catalysts. Ag particles were found to be more outside for the low surface carbon, while the majority was also found inside for the mesoporous high surface carbon Ketjenblack.

However, in electrochemical characterization in a gas diffusion electrode of a zero-gap electrolyzer cell, all three carbon supported Ag catalysts showed similar performance. This is a strong indicator that the peak performance and the mass activity are not limited by the catalyst kinetics but by mass transport limitations similarly affecting all three supported catalysts. This further means that the catalyst support surface area does not play a role in the investigated systems, which used PTFE as an electrode binder. This is in strong contrast to fuel cell electrodes, where the surface area of the carbon support and the resulting uptake of ion-conductive binder has a strong effect on performance and durability.^33^ With this, one could assume that when using an anion conductive electrode binder, which could be beneficial especially with increasing water consumption at high current densities (*e.g.*, > 200 mA cm^−2^), the surface area of the carbon could play an important role.

Compared to literature the here synthesized catalysts show outstanding mass activities for AEM-based CO_2_ reduction under real operating conditions, with a maximum Ag mass activity up to 3920 mA mg^−1^ at 3.2 V in 0.1 M KOH and 5400 mA mg^−1^ at 3.3 V with 0.1 M CsOH, outperforming reported Ag and Au supported catalysts. The results of our study indicate that the catalyst is not a limiting factor in cell measurements. Specifically, we observed stable FE_CO_ greater than 80% for over 11 h at 300 mA cm^−2^ in 0.1 M CsOH, with no significant degradation of Ag/C catalysts. These findings suggest thiol-based linkers as promising candidates for achieving long-term durability. To further increase the activity, the wettability and transport properties of the electrode need to be controlled *via* tailoring hydrophobicity and particle sizes of the additive (binder, ionomer, linker and catalyst support). Furthermore, increasing the Ag/C loading or the Ag loading on carbon may result in higher current densities. Such optimization will provide further insight into the distinction between the three Ag/C catalysts.

## Conflicts of interest

The authors declare no competing financial interest.

## Supplementary Material

RA-013-D3RA03424K-s001

RA-013-D3RA03424K-s002

RA-013-D3RA03424K-s003

RA-013-D3RA03424K-s004

RA-013-D3RA03424K-s005

RA-013-D3RA03424K-s006

RA-013-D3RA03424K-s007
