# Supplementary material for: Carbon black supported Ag nanoparticles in zero-gap CO2 electrolysis to CO enabling high mass activity
Source: RSC Adv. 2023 Jun 21;13(27):18916–26. doi: 10.1039/d3ra03424k (PMC10283028; doi:10.1039/d3ra03424k)
Supplement: RA-013-D3RA03424K-s007 [file RA-013-D3RA03424K-s007.pdf]

Electronic Supplementary Material (ESI) for RSC Advances.
This journal is © The Royal Society of Chemistry 2023

Carbon black supported Ag nanoparticles in zero-gap CO_2 electrolysis to CO enabling high mass activity

*Khaled Seteiz[a,b‡], Josephine N. Häberlein[a,b‡], Philipp A. Heizmann[a,b], Joey Disch[a,b] and Severin Vierrath[a,b,c]**

[a]Electrochemical Energy Systems, IMTEK - Department of Microsystems Engineering, University of Freiburg, Georges-Koehler-Allee 103, 79110 Freiburg, Germany

[b]University of Freiburg, Institute and FIT – Freiburg Center for Interactive Materials and Bioinspired Technologies, Georges-Köhler-Allee 105, 79110 Freiburg

[c]Hahn-Schickard, Georges-Koehler-Allee 103, 79110 Freiburg, Germany

[‡]authors contributed equally

*corresponding author

Contents

Figure S1: Synthesis scheme of S-anchored Ag/C catalysts. 3
Figure S2: Mapped Ag loading determined from XRF measurements on GDEs. 4
Figure S3: Casted $NiFe_2O_4$ HCCM and casted IrO_2 HCCM. 5
Figure S4: TGA of Ag/C catalysts. 6
Figure S5: Assembly of cell components in zero-gap CO2 electrolyzer. 7
Figure S6: TEM images of pure carbon support Super P. 8
Figure S7: TEM images of pure carbon support Vulcan. 9
Figure S8: TEM images of pure carbon support Ketjenblack. 10
Figure S9: EDX: HAADF micrograph. 12
Figure S10: EDX Spectra of $Ag/C_{Super\ P}$, Ag/C_{Vulcan} and Ag/C_{Ketjen}. 13
Figure S11: EDX Spectra of Super P, Vulcan and Ketjenblack. 14
Figure S12: EDX Spectra of commercial Ag/C catalysts. 15
Figure S13: Fourier integral analysis of a representative HRTEM micrograph. 16
Figure S14: Ag particle size distribution: $Ag/C_{Super\ P}$. 17
Figure S15: Ag particle size distribution: Ag/C_{Vulcan}. 18
Figure S16: Ag particle size distribution: Ag/C_{Ketjen}. 19
Figure S17: Ag particle size distribution. Overlap of Ag/C_{Ketjen}, Ag/C_{Vulcan} and $Ag/C_{Super\ P}$. 20
Figure S18: Selected projections from a STEM tilt series. 21
Figure S19: Selected projections from a STEM tilt series of an $Ag/C_{Super\ P}$ sample 22
Figure S20: Average Raman spectra. 23
Figure S21: Lorentz plot of Raman spectra 24
Figure S22: Pb-UPD of the Ag/C catalysts and pristine H23C6 GDL. 25
Figure S23: EDLC measurement of the Ag/C catalysts and pristine H23C6 GDL. 26
Figure S24: Contact angle of DI water. 27
Figure S25: Additional cell performances of Ag/C catalysts. 28
Figure S26: Cell performance with Carbon black Super P. 29

Figure S27: CO and H_2 Faradaic efficiencies of Ag/C catalysts..30
Figure S28: Cell voltage in terms of CO partial current density of commercial Ag/C. 31
Figure S29: TEM images of commercial 40 % Ag/C_{Vulcan}. 32
Figure S30: TEM images of commercial 80 % Ag/C_{Vulcan}. 33
Figure S31: pH change after long-term operation. 34
Figure S32: Ni-felt degradation after long-term experiments. 35
Figure S33: Post-mortem TEM images after long-term operation. 35

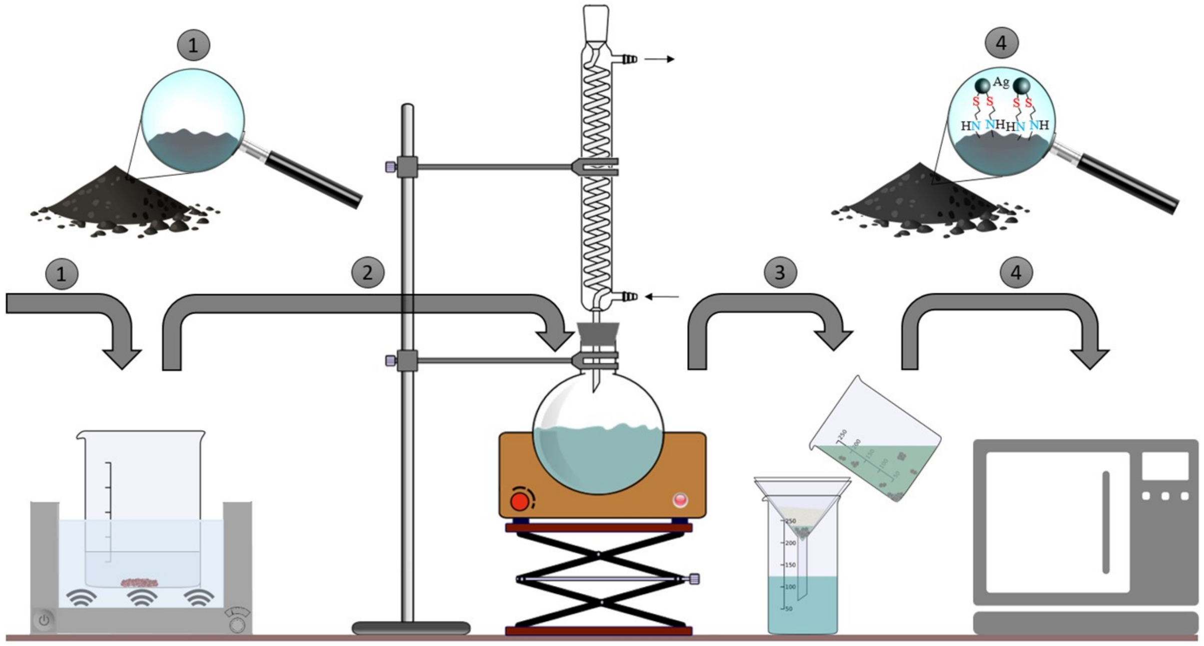

Figure S1: Synthesis scheme of S-anchored Ag/C catalysts. **(1)**: Carbon support (200 mg) in EG (100 ml) ultrasonicated for 30 min. Cysteamine (10 mg) added to carbon support solution and ultrasonicated for another 30 min **(2)**: Mixture added to preheated (50 °C) solution of $AgNO_3$ (200 mg) in EG (100 ml) and heated to 180°C under reflux and kept for 60 min **(3)**: Filtration of mixture and washed with isopropanol **(4)**: Drying overnight at 80 °C. Ag/C catalysts were obtained as black powder.

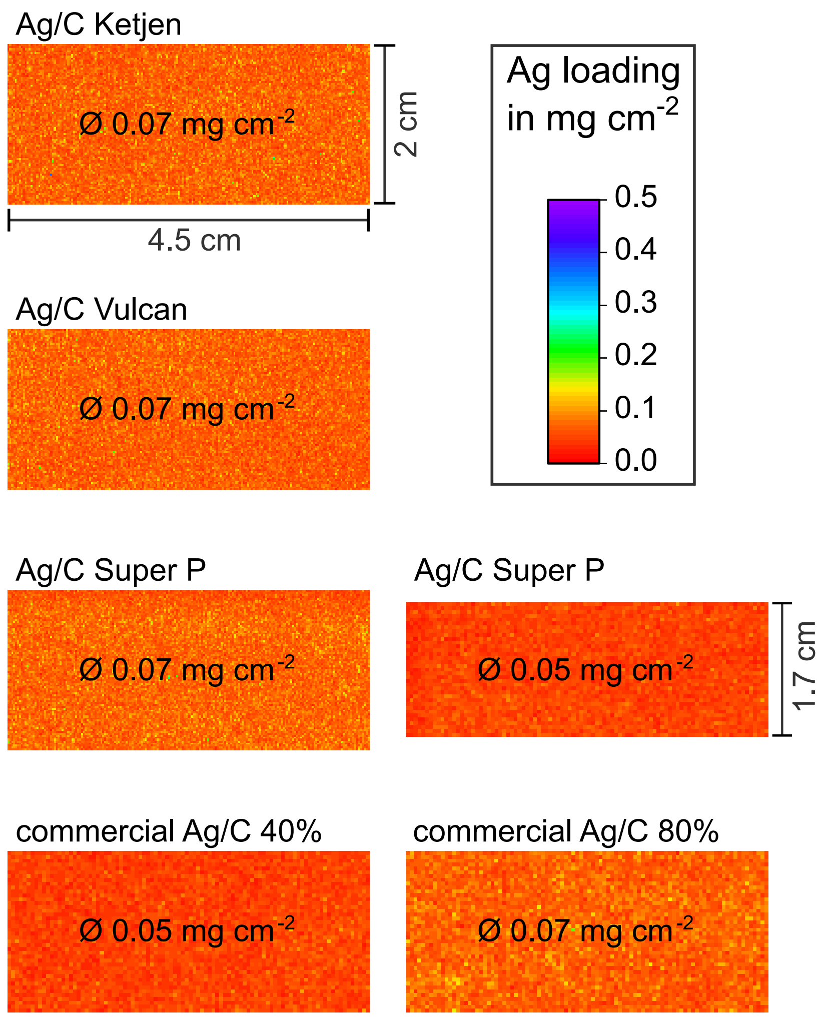

Figure S2: Mapped Ag loading determined from XRF measurements on GDEs. The procedure of measurement was the same for each mapping, but the size of the pixel differed between the two series of measurements.

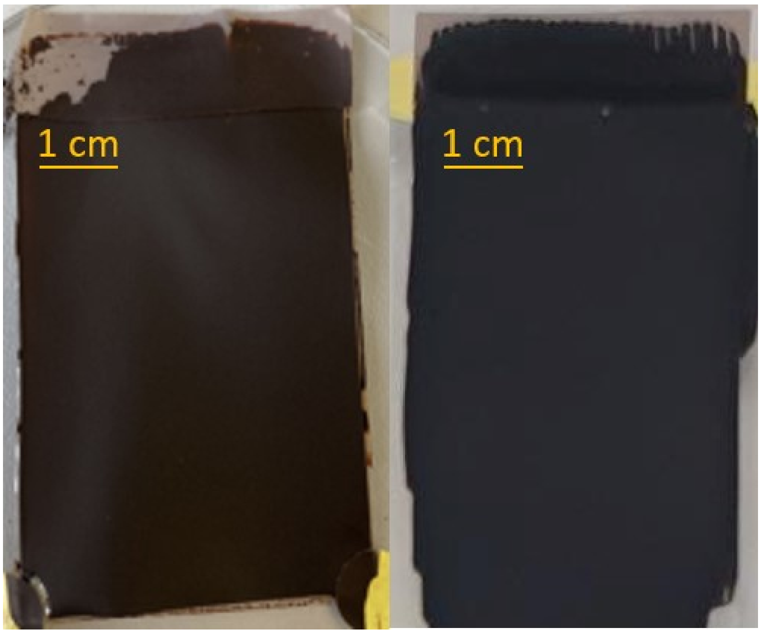

Figure S3: Casted $NiFe_2O_4$ HCCM (left) and casted IrO_2 HCCM (right).

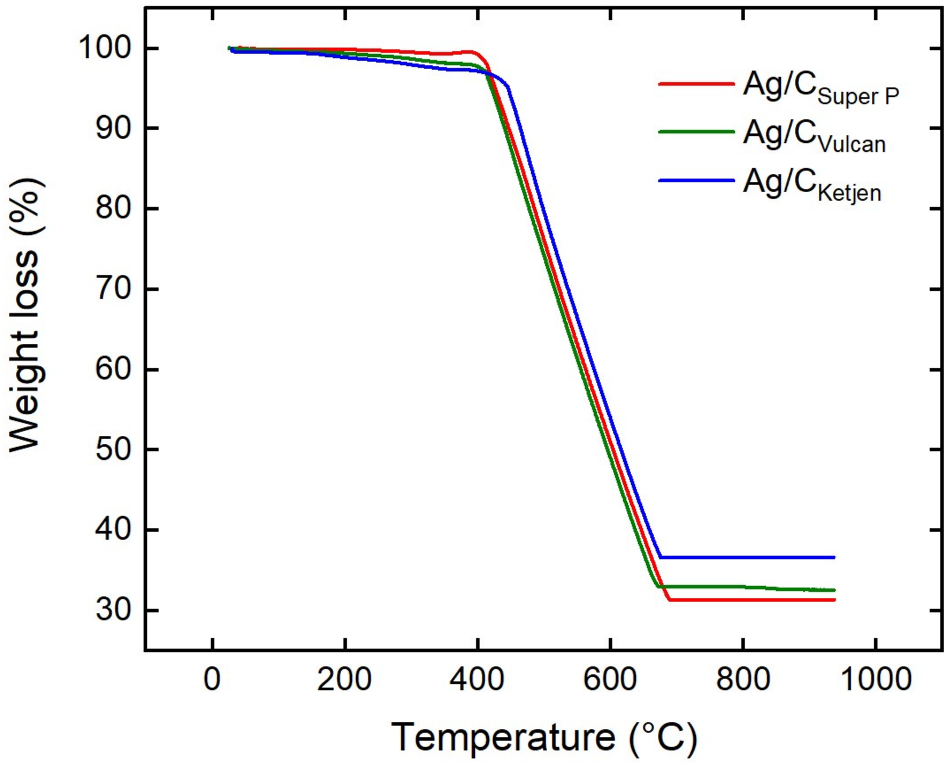

Figure S4: TGA of Ag/C catalysts under oxygen atmosphere with a heating rate of 10 °C min^{-1} and flow rate of 100 ml min^{-1}.

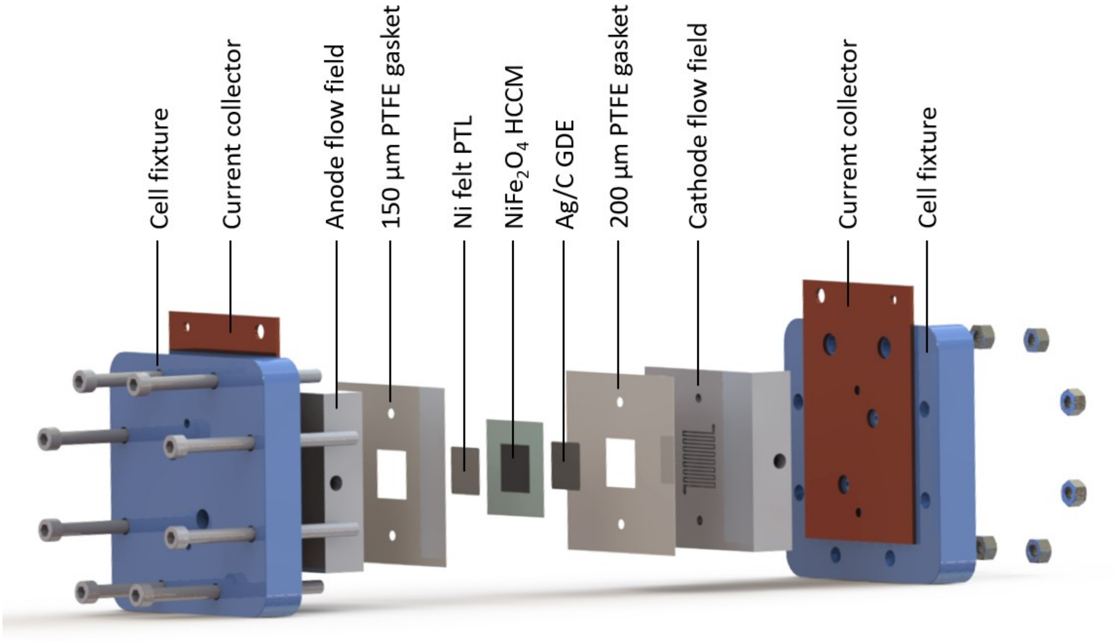

Figure S5: Assembly of cell components in zero-gap CO2 electrolyzer.

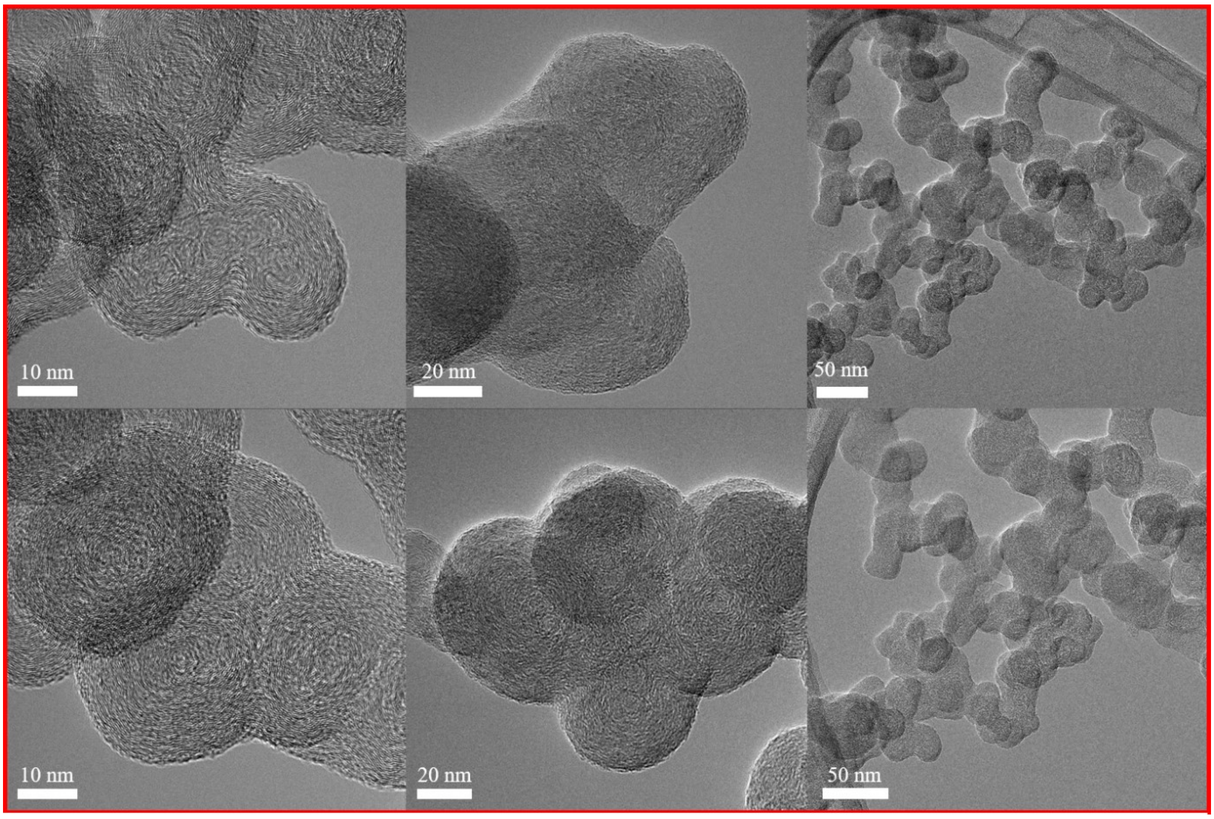

Figure S6: TEM images of pure carbon support Super P® conductive (further denoted as Super P).

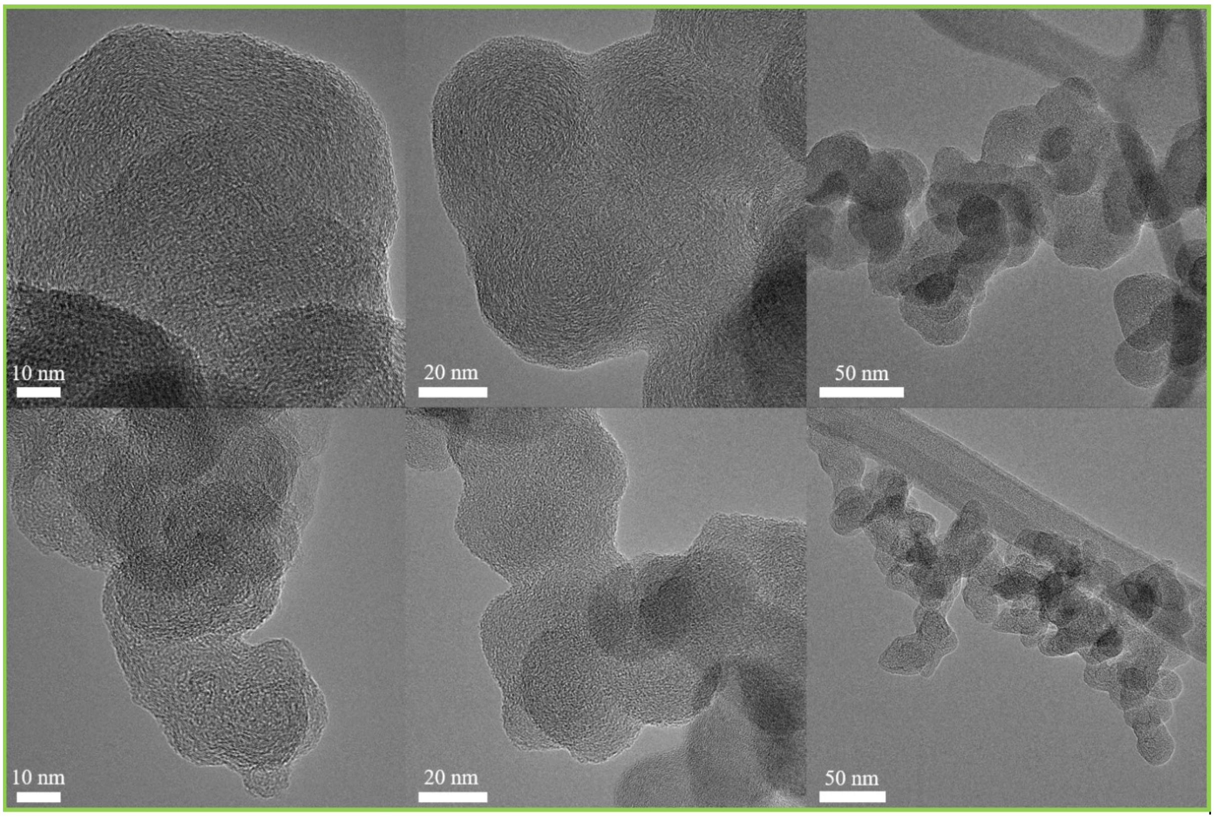

Figure S7: TEM images of pure carbon support Vulcan XC-72-R (further denoted as Vulcan).

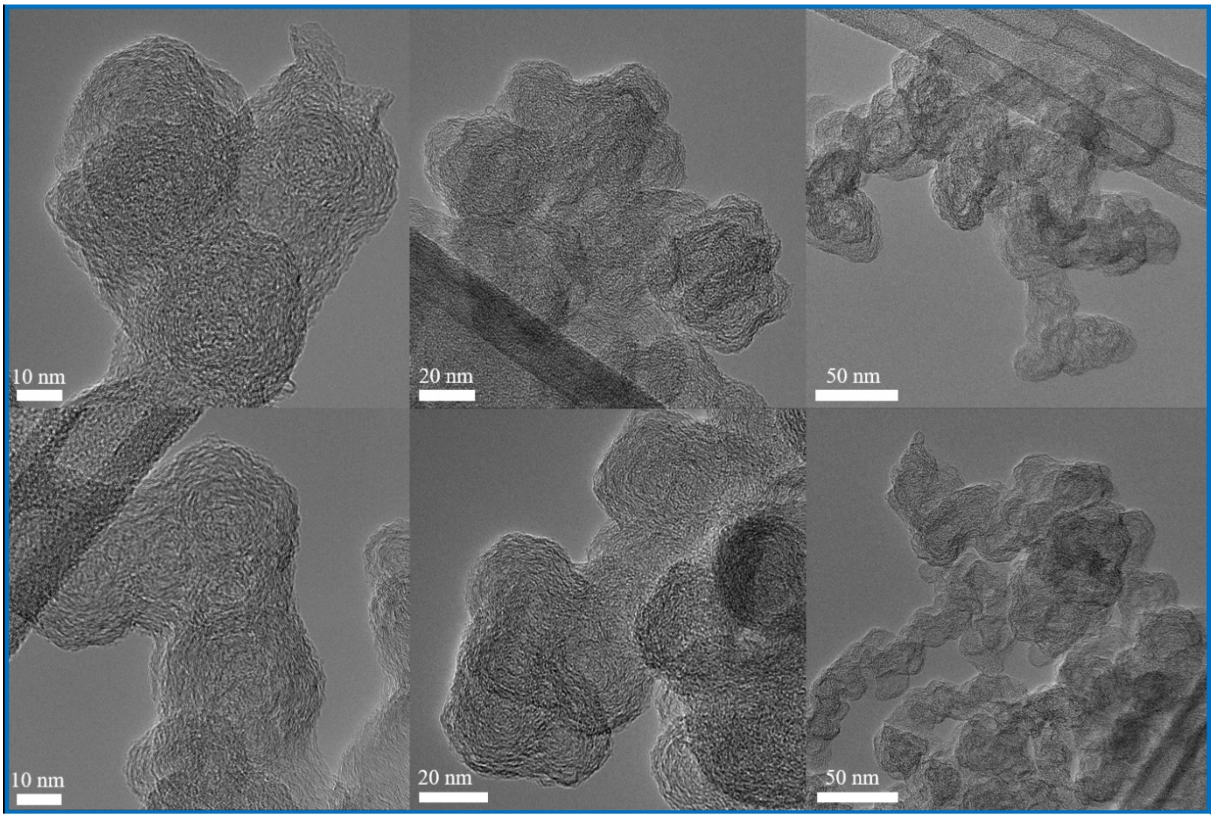

Figure S8: TEM images of pure carbon support Ketjenblack EC-300J (further denoted as Ketjenblack).

EDX-analysis

The elemental nature of the Ag nanoparticles is demonstrated via EDX mapping (Figure S9). Normalized to the carbon Kα peak, a peak intensity decrease in the other element unique emissions in the order $Ag/C_{Super\ P}$ > Ag/C_{Vulcan} > Ag/C_{Ketjen} can be observed, another strong indication of the solid nature of the Super P sample compared to the more hollow nature of the Ketjenblack carbon species as surface-based signals are enhanced at reduced sample thickness. Sulfur could be detected on all samples, a further confirmation of the anchor-type reaction scheme as proposed by Kim *et al.*.[1]

In case of Vulcan, sulfur was already present in the sample prior to the nanoparticle synthesis, but a strong increase of the sulfur peak was observable after the Ag/C product was obtained. The respective elemental mappings of sulfur suggests an accumulation of sulfur at the positions of silver nanoparticles. Copper and silicon was also detected in all samples. While the former stems from the copper based TEM grid, the latter was likely introduced to the samples during the synthesis in glass vessels as EDX spectra of the as-received carbon supports show none or significantly reduced amounts of trace Si (Figure S11). Interestingly, both commercial Ag/C species contain traces of chlorine, presumably indicating that these catalysts were obtained by reduction of a silver chloride precursor species onto the carbon support (Figure S12).

Fourier integral analysis of HRTEM images of the synthesized Ag/C yielded four distinctive repeating periodic patterns with spacings of ~0.23 nm, ~0.20 nm, ~0.14 nm and ~0.11 nm (Figure S 13). These values closely match with reported interplanar distances of face centered cubic (FCC) Ag at 0.2355 nm (miller indices hkl 111), 0.2039 nm (hkl 200), 0.1442 nm (hkl 220) and 0.1177 nm (hkl 311).[2,3]

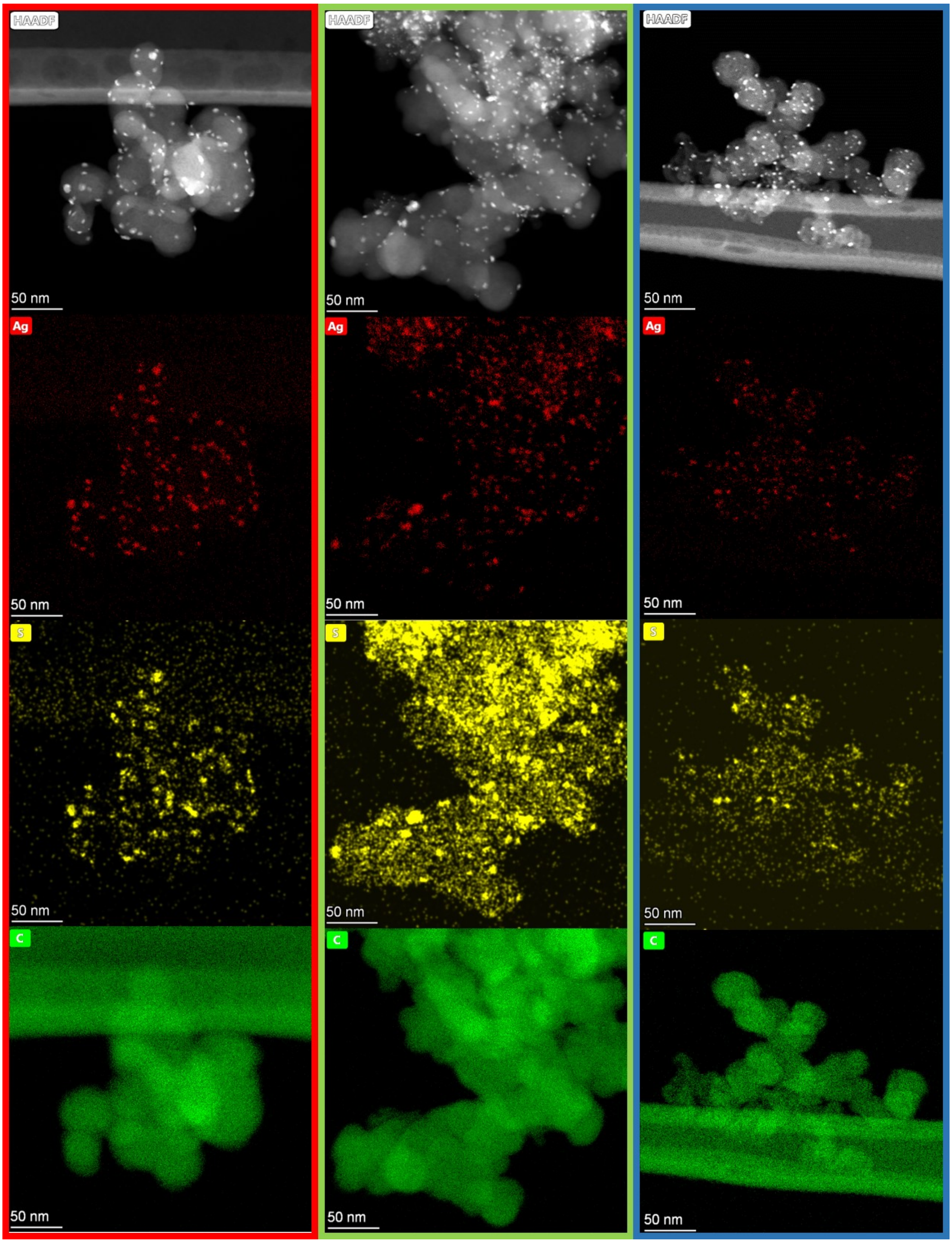

Figure S9: EDX: HAADF micrograph, C (green), S (yellow) und Ag (red) elemental distributions of Ag/$C_{Super\ P}$ (red), Ag/C_{Vulcan} (green) and Ag/C_{Ketjen} (blue). Due to a comparatively low number of counts, the elemental maps for sulfur were enhanced for better visibility.

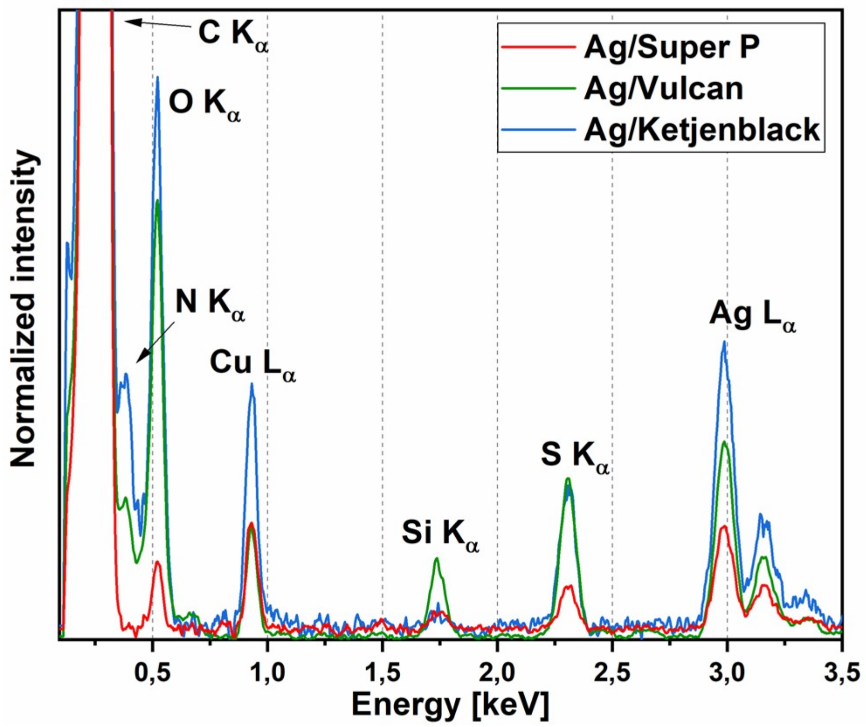

Figure S10: EDX Spectra of Ag/$C_{Super\ P}$, Ag/C_{Vulcan} and Ag/C_{Ketjen}, normalized to the C Kα signal. The Cu Lα signal stems from the copper based TEM grid.

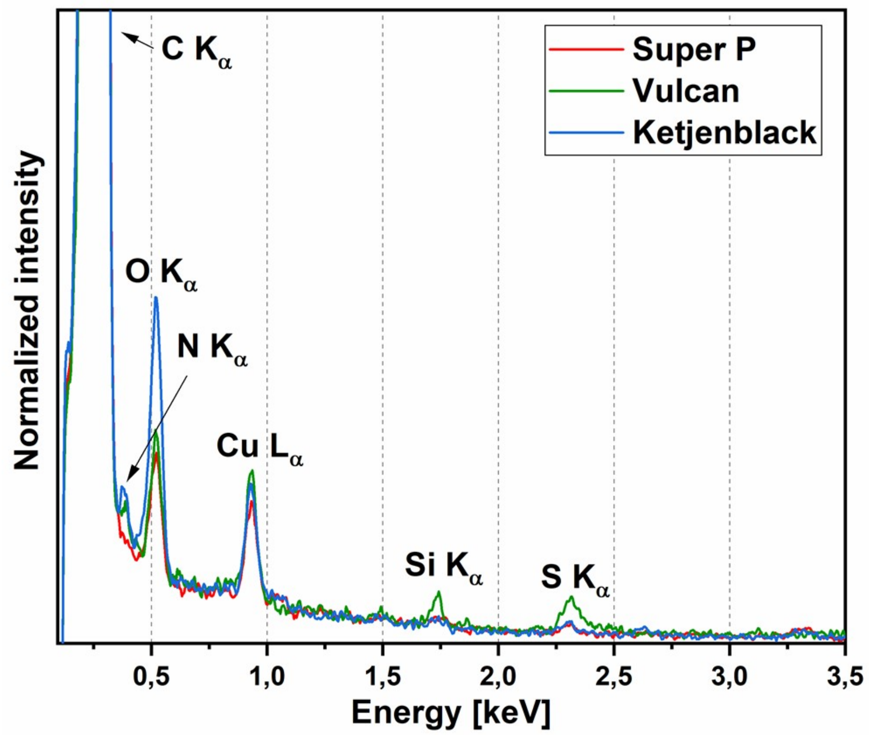

Figure S11: EDX Spectra of Super P, Vulcan and Ketjenblack, normalized to the C Kα signal. The Cu Lα signal stems from the copper based TEM grid.

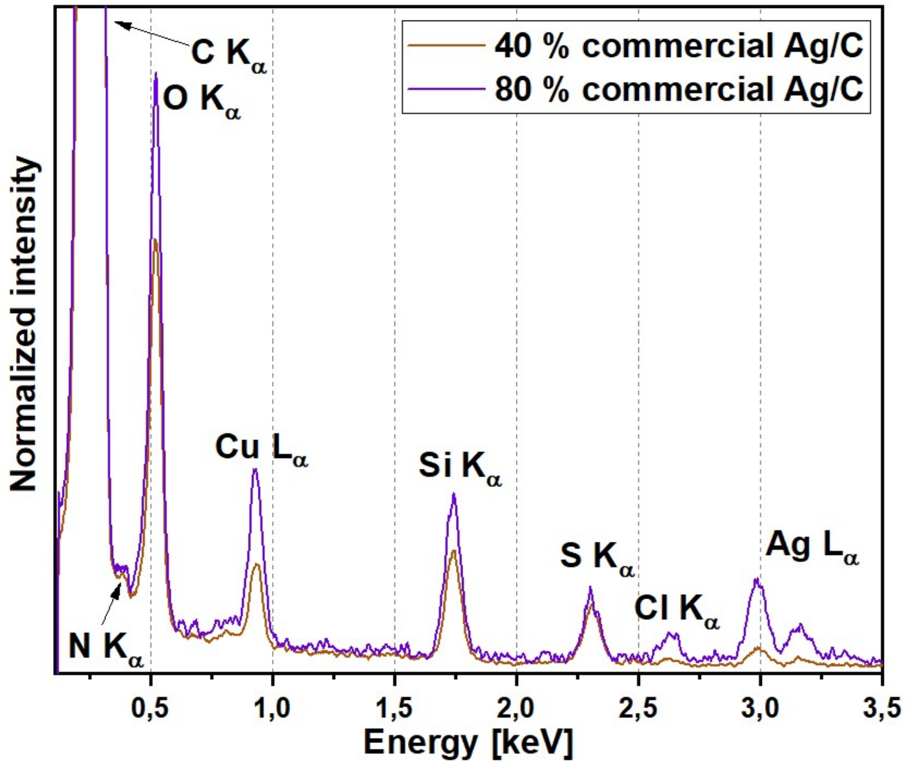

Figure S12: EDX Spectra of commercial Ag/C catalysts, normalized to the C Kα signal. The Cu Lα signal stems from the copper based TEM grid.

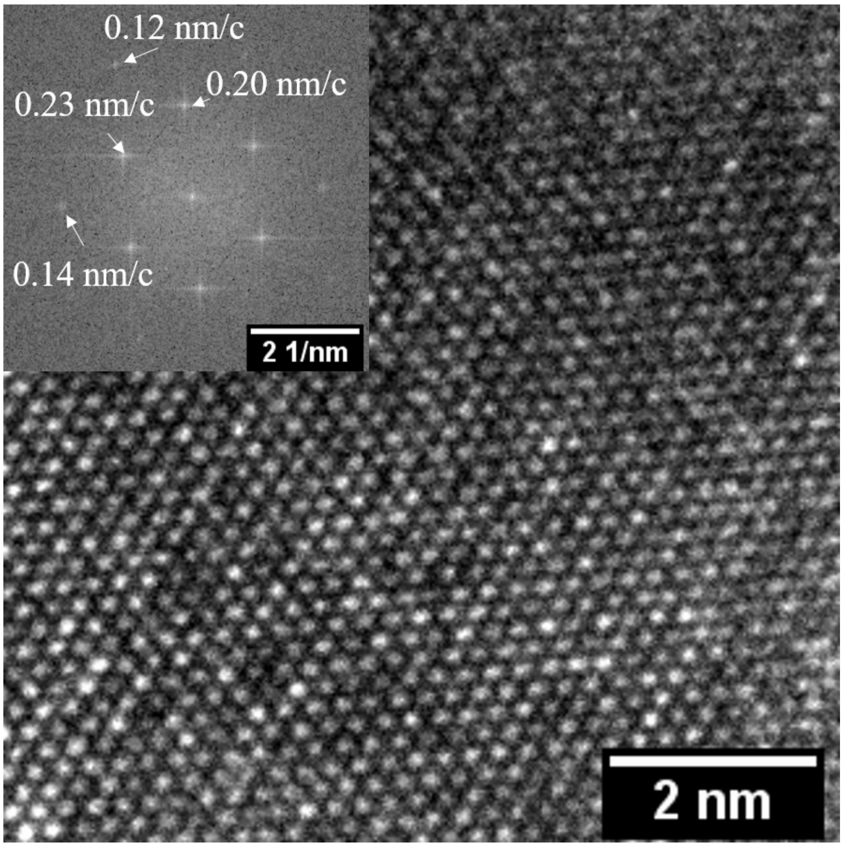

Figure S13: Fourier integral analysis of a representative HRTEM micrograph of an individual Ag nanoparticle.

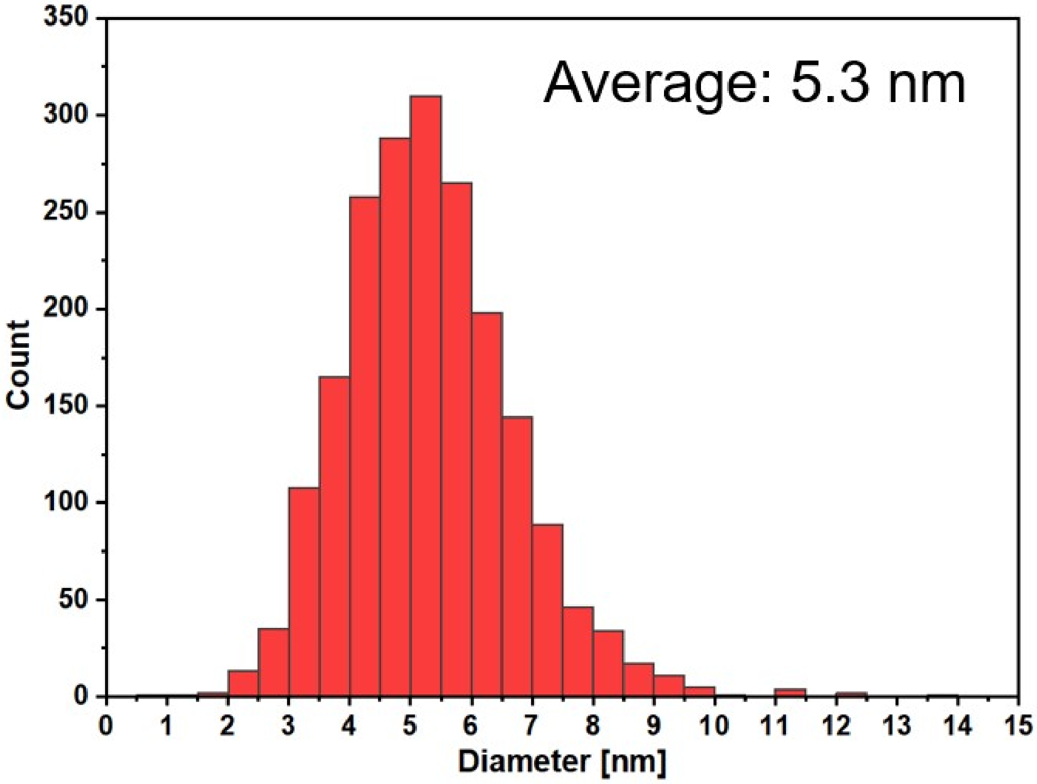

Figure S14: Ag particle size distribution: Ag/$C_{Super\ P}$.

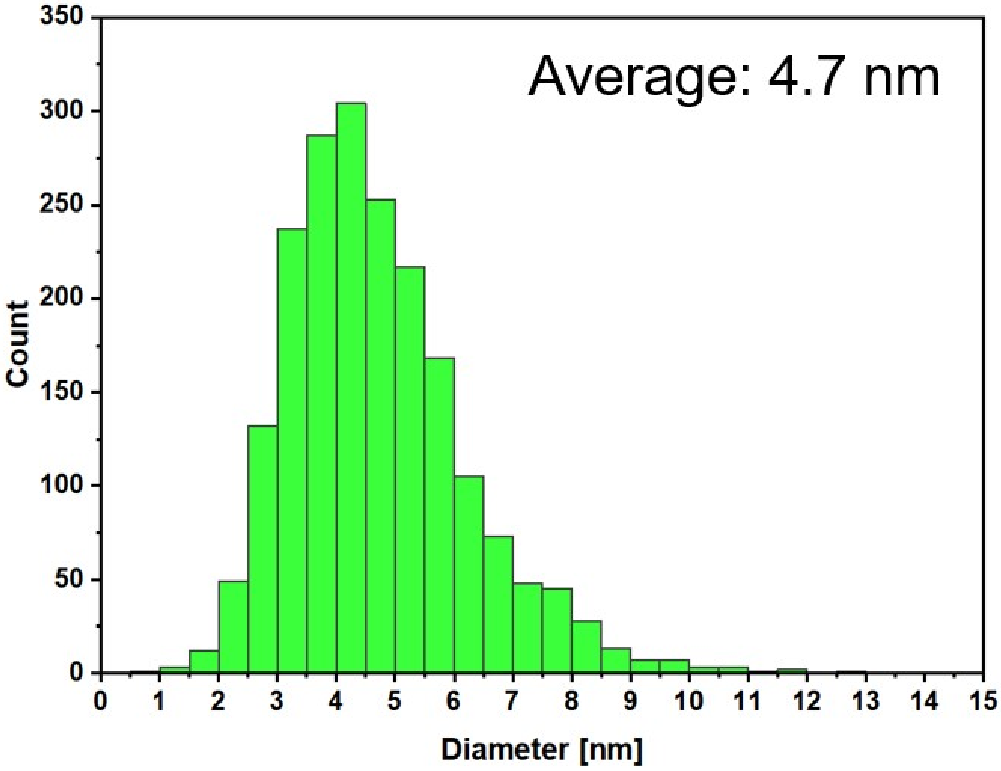

Figure S15: Ag particle size distribution: Ag/C_{Vulcan}.

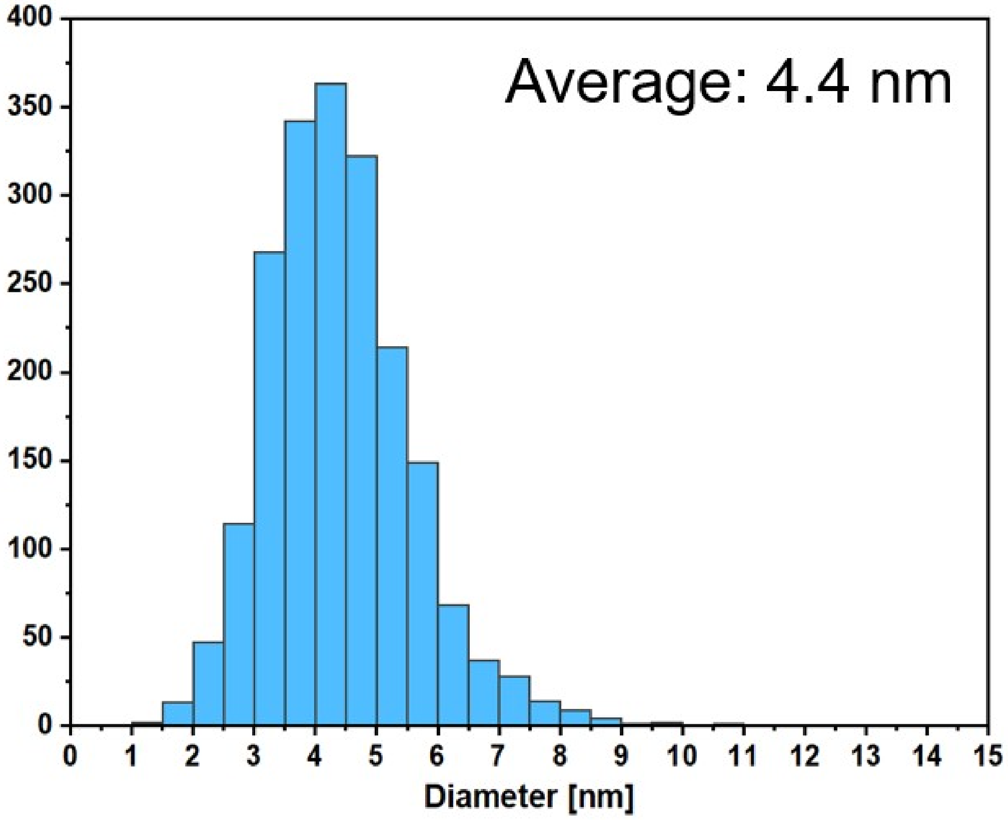

Figure S16: Ag particle size distribution: Ag/C_{Ketjen}.

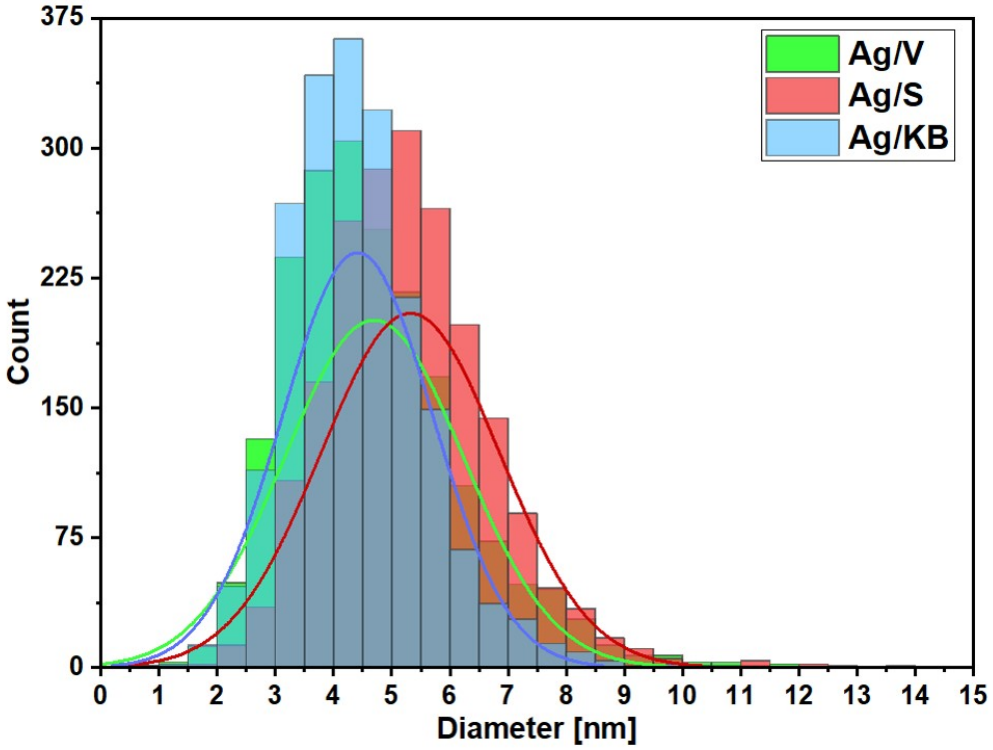

Figure S17: Ag particle size distribution. Overlap of Ag/C$_{Ketjen}$ (blue), Ag/C$_{Vulcan}$ (green) and Ag/C$_{Super\ P}$ (red).

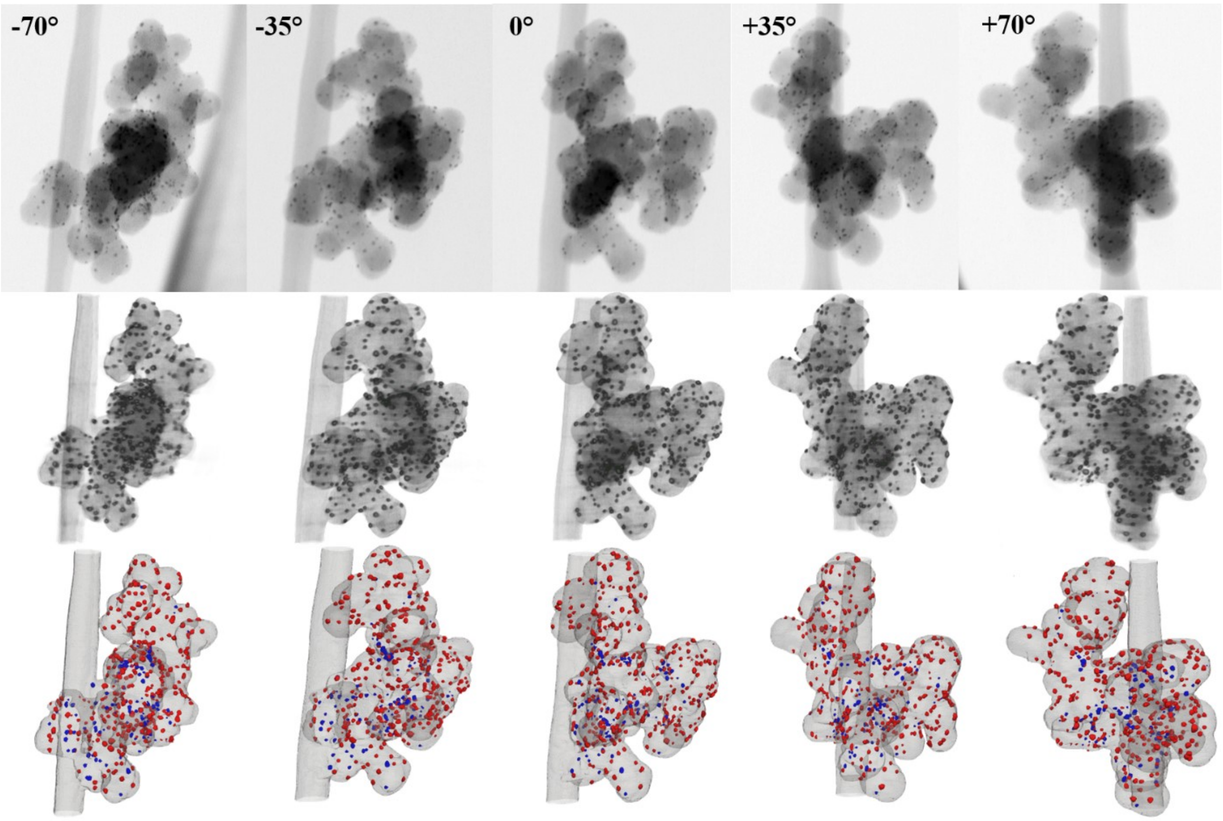

Figure S18: Selected projections from a STEM tilt series (BF mode) of an Ag/C_{Vulcan} sample. Upper row: Raw 2D STEM images at specified tilt angles. Middle row: Reconstructed volumes. Lower row: Segmented volumes (red: exterior nanoparticles, blue: interior nanoparticles). Note that both the reconstructed and segmented volumes contain depth.

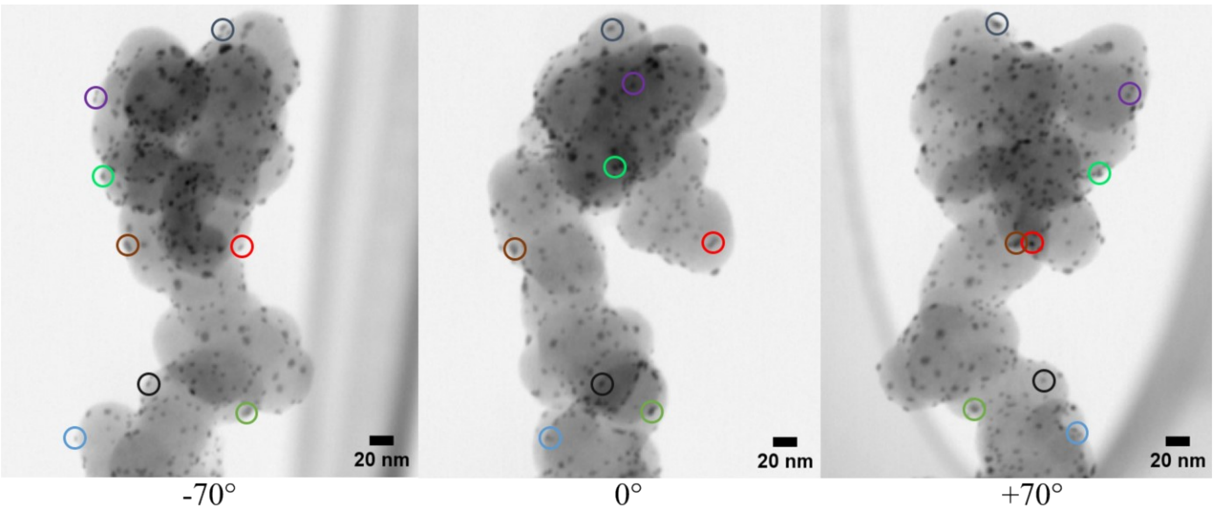

Figure S19: Selected projections from a STEM tilt series (BF mode) of an Ag/$C_{Super\ P}$ sample. The circles mark particles whose position (interior or exterior) cannot be determined from the 0° projection, but which can be identified as exterior particles at other tilt angles within the tilt series.

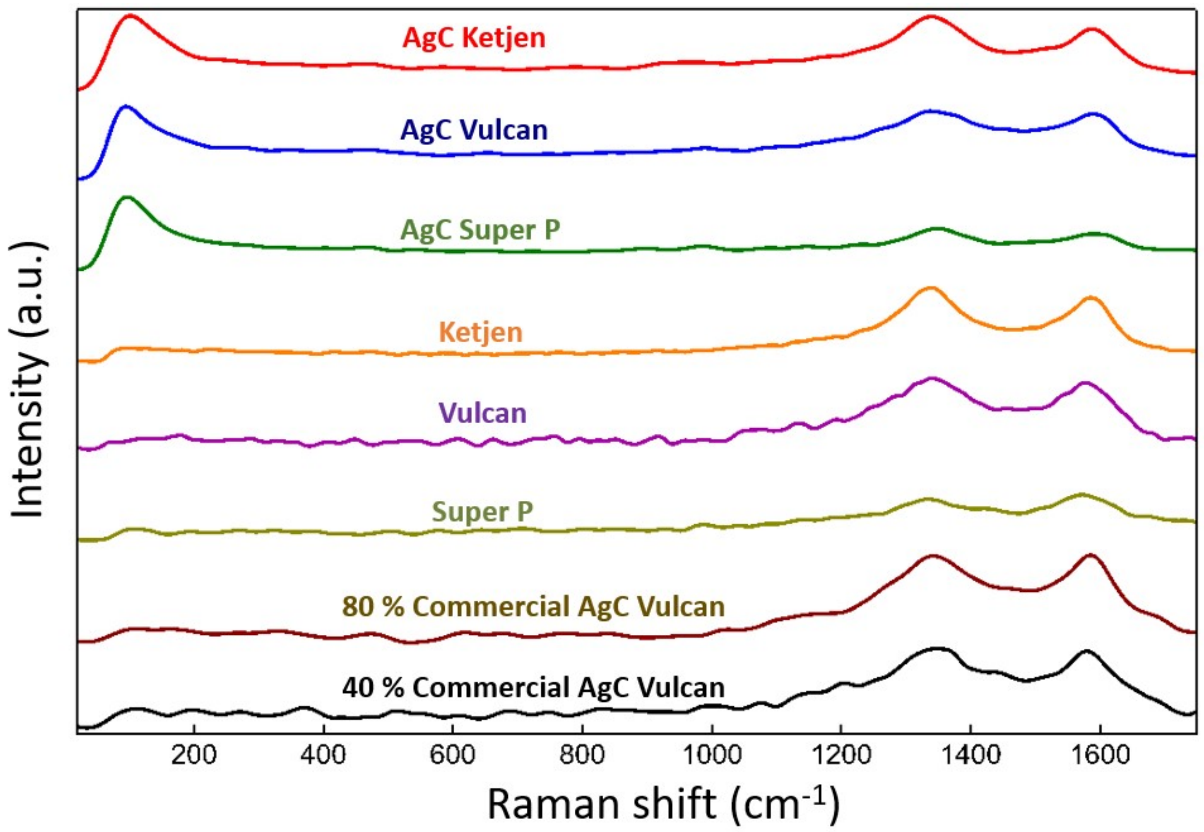

Figure S20: Average Raman spectra of Ag/C_{Ketjen}, T Ag/C_{Vulcan}, $Ag/C_{Super\ P}$, Ketjenblack, Vulcan, Super P, commercial 80 % Ag/C_{Vulcan} and commercial 40 % Ag/C_{Vulcan}. Marginal trend in defect concentration. (Ratio D band to G band) in the order Ketjen > Vulcan > Super P. Significant Ag–S bond formation with Ag/C_{Ketjen}, Ag/C_{Vulcan} and $Ag/C_{Super\ P}$.

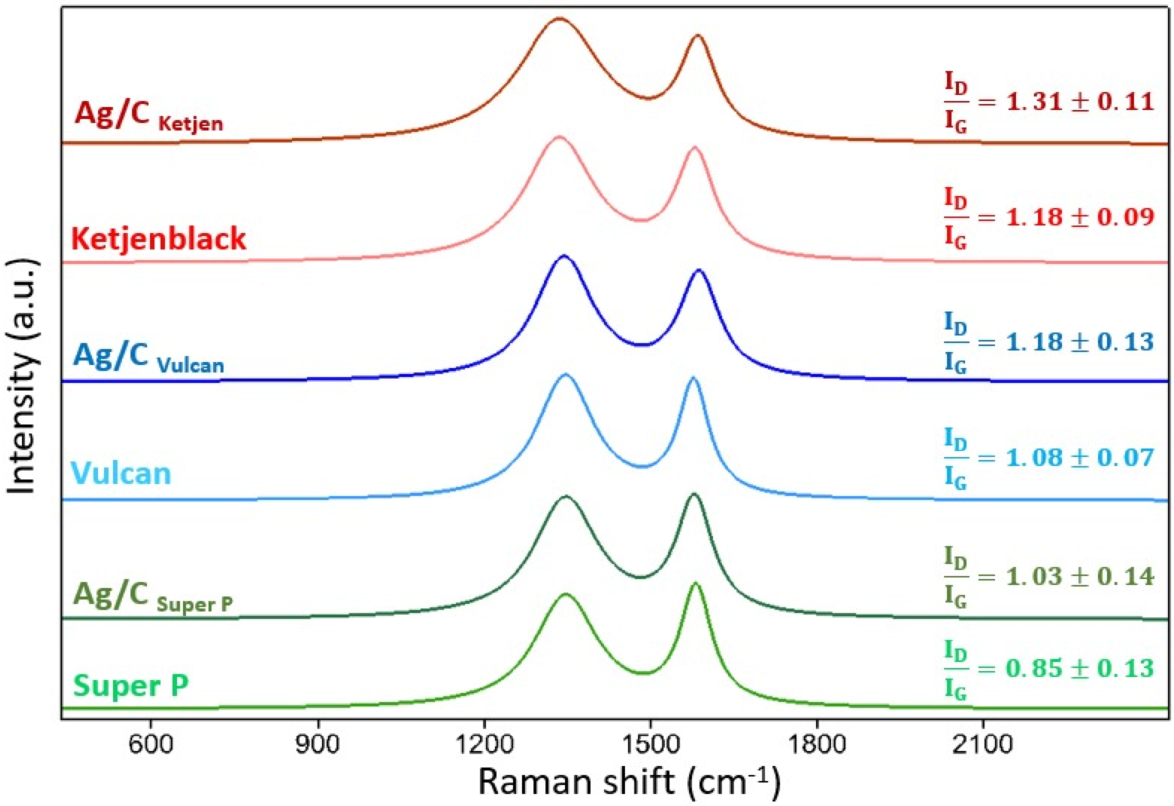

Figure S21: Lorentz plot of Raman spectra Figure S20. The ratio of the D band and G band intensities (I_D/I_G) were obtained by dividing the amplitude of the D-band by the amplitude of G-band after applying a Lorentz fit. The error in the I_D/I_G ratio was calculated using error propagation with the values obtained from the Lorentz fit. The following trend can be observed when comparing carbon blacks: Ketjenblack has the highest defect concentration (I_D/I_G ratio of 1.18, followed by Vulcan (ratio of 1.08), and then Super P (ratio of 0.85).With Ag/C_{Ketjen}, Ag/C_{Vulcan} and Ag/$C_{Super\ P}$ the ratio is 1.31, 1.18, and 1.03, respectively.[4],[5]

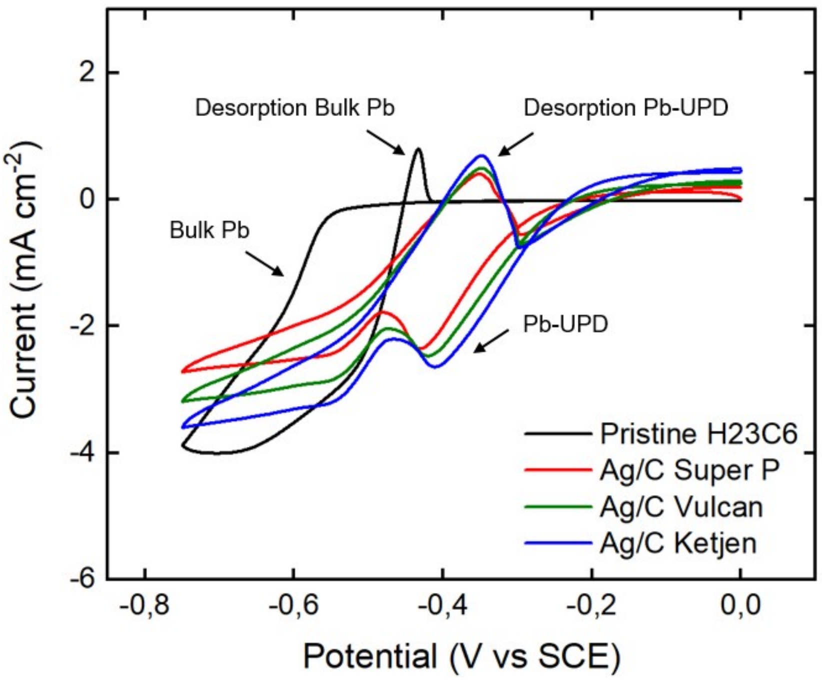

Figure S22: Pb-UPD of the Ag/C catalysts and Pristine H23C6 GDL. CV was performed in a potential range of –0.8 to 0 V vs. SCE at a scan rate 5 mV/s in 1 mM Pb(acetate)$_2$ + 1 mM $HClO_4$ + 0.5 M $NaClO_4$ solution.

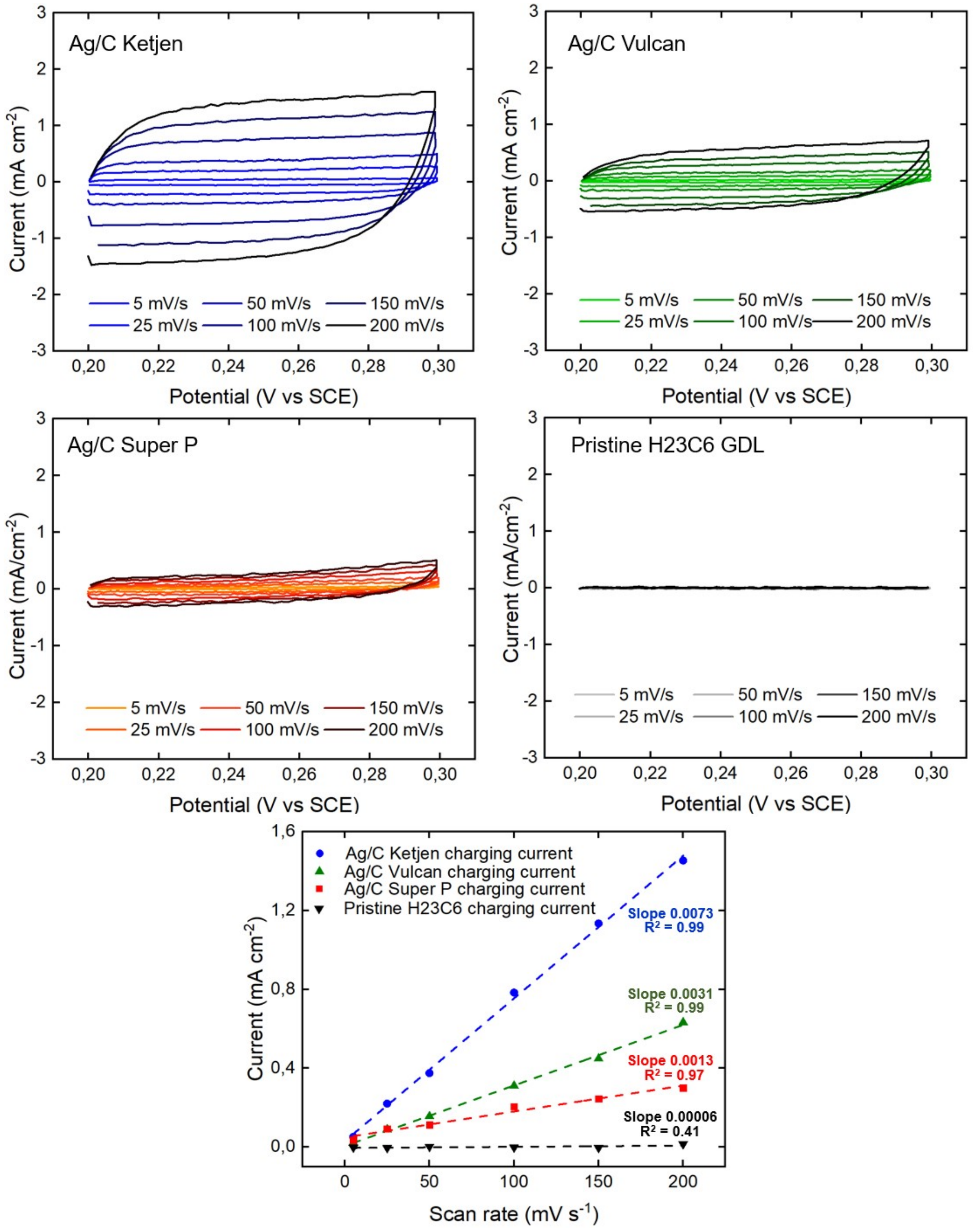

Figure S23: EDLC measurement of the Ag/C catalysts and pristine H23C6 GDL. CV was performed in a potential range of 0.2 to 0.3 V vs. SCE with different scan rates (v = 5, 25, 50, 100, 150 and 200 mV/s) in 1 M H_2SO_4. The peak currents of GDEs were plotted against scan rates and fitted linearly.

Water contact angle measurements

To determine whether the different electrodes were hydrophobic or hydrophilic, the water contact angles for the Ag/C GDEs and the pristine GDL were measured. All of the electrodes were found to be hydrophobic, with the pristine GDL having the highest water contact angle of 144°, followed by Ag/$C_{Super\ P}$ (127°), Ag/C_{Vulcan} (117°), and Ag/C_{Ketjen} (103°). The water contact angle of the pristine H23C6 GDL is similar to that of commercial GDLs, ranging from 135° to 148°.[6]

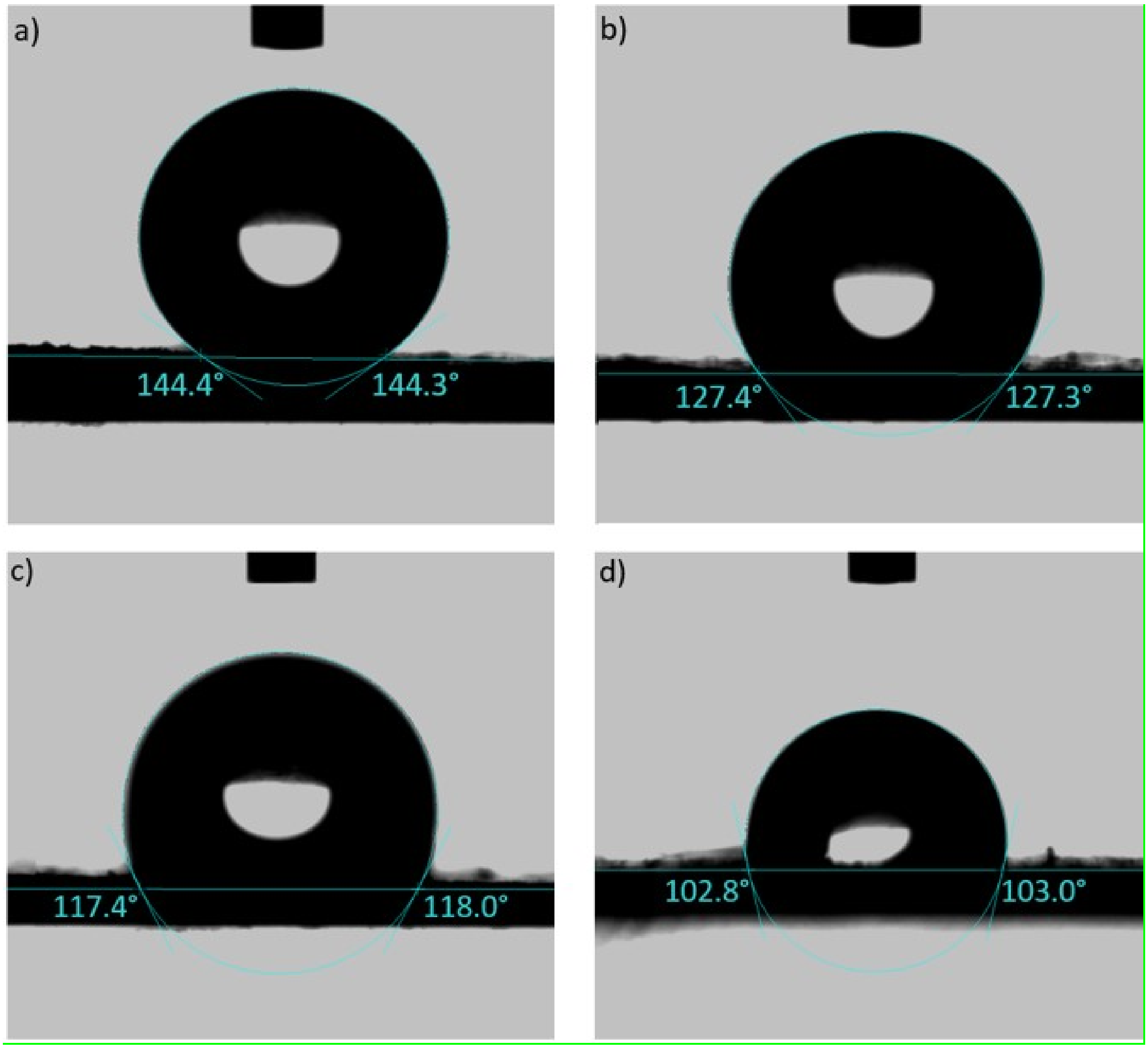

Figure S24: Contact angle of DI water on the a) pristine H23C6 GDL, b) Ag/$C_{Super\ P}$ GDE, c) Ag/C_{Vulcan} GDE and d) Ag/C_{Ketjen} GDE. Sample sizes of 2 cm^2 were used and each water droplet volume is 5 µL.

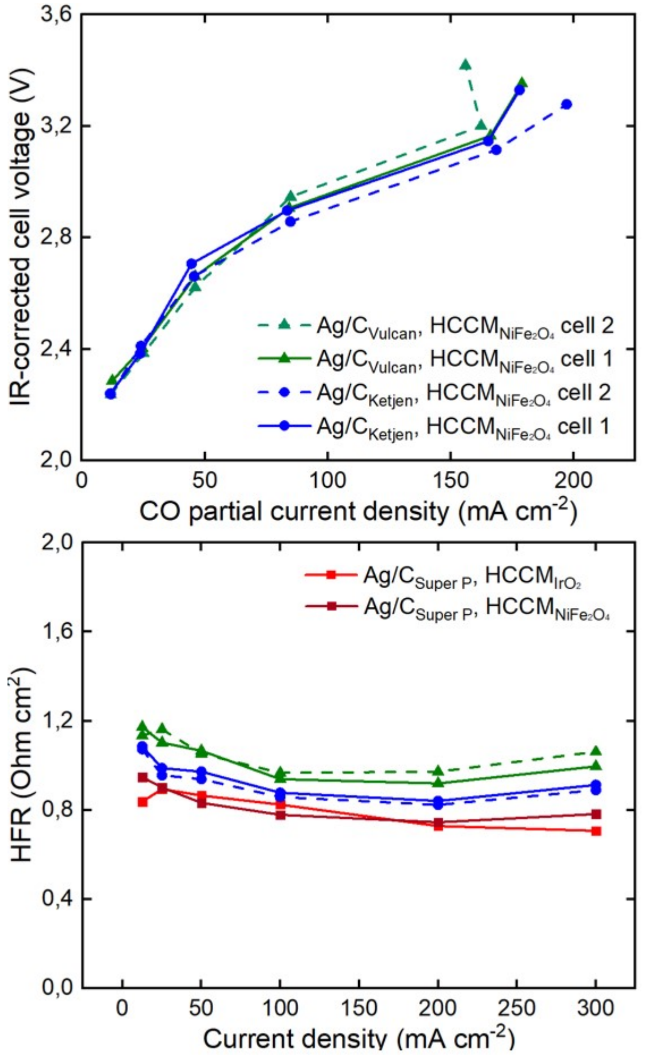

Figure S25: A) Additional cell performances of Ag/C catalysts and b) respective HFR.

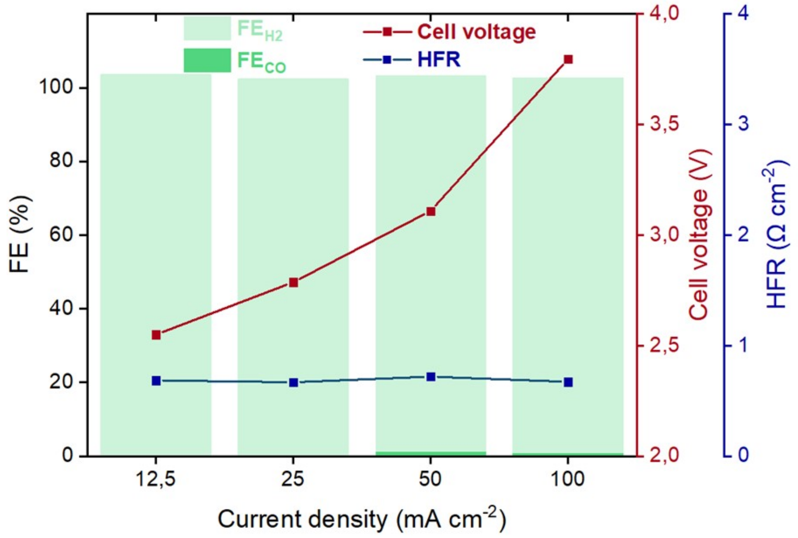

Figure S26: Cell performance with Carbon black Super P (without Ag) as cathode catalyst in 0.1 M KOH with IrO_2 HCCM. The results demonstrate $j_{CO,\,max}$ = 0.84 $mAcm^{-2}$ (when applying 100 $mAcm^{-2}$ to the cell) at 3.78 V with Super P. As only negligible formation of CO with concomitant low overall FE_{CO} were observed by gas chromatography, the high selectivity and increase in mass activity is thus mainly ascribed to Ag on the Ag/C catalysts, respectively.

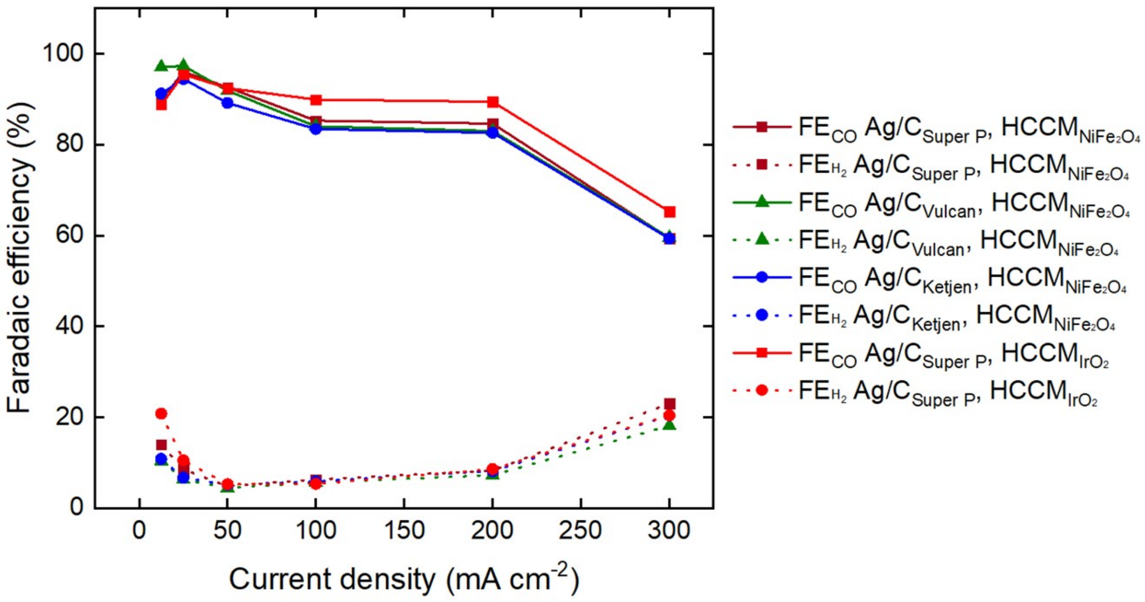

Figure S27: CO and H_2 Faradaic efficiencies of Ag/C catalysts.

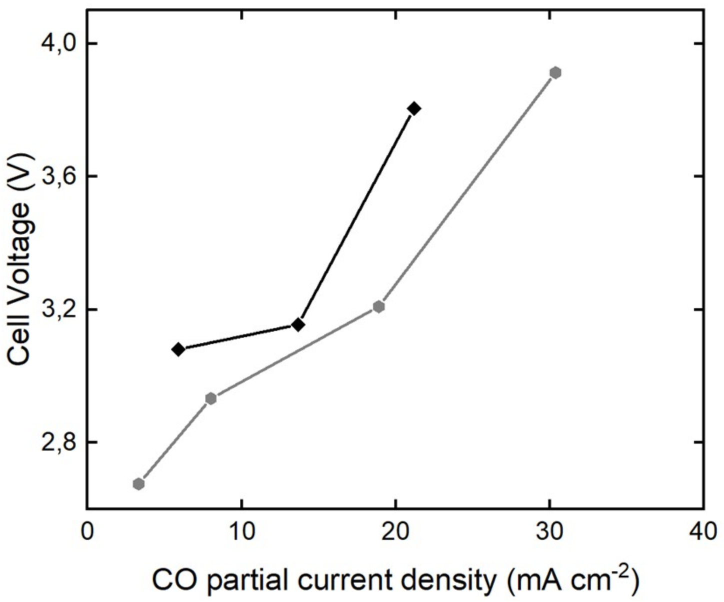

Figure S28: Cell voltage in terms of CO partial current density of commercial 80 % Ag/C_{Vulcan} (grey) and commercial 40 % Ag/C_{Vulcan} (black).

TEM images of commercial Ag/Cs

Compared to the in-house synthesized Ag/C, the commercial Ag/C_{Vulcan} catalysts are characterized by very large (> 100 nm) Ag nanoparticles that lie loosely and chemically unbound on the carbon support (Figure S29). As the silver particles are not chemically bound to the carbon and are too large to sit within the carbon pores, electron tomography was not required. From geometrical considerations alone, it is to be expected that these catalysts show significantly poorer performance due to the substantially lower available Ag surface.

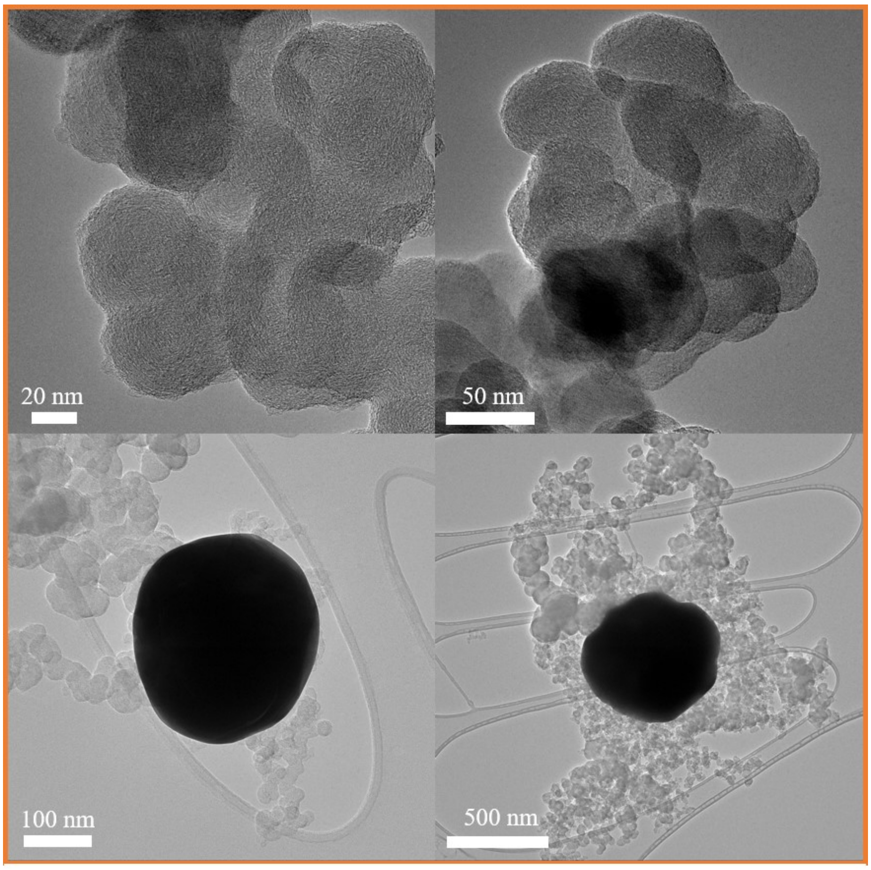

Figure S29: TEM images of commercial 40 % Ag/C_{Vulcan}.

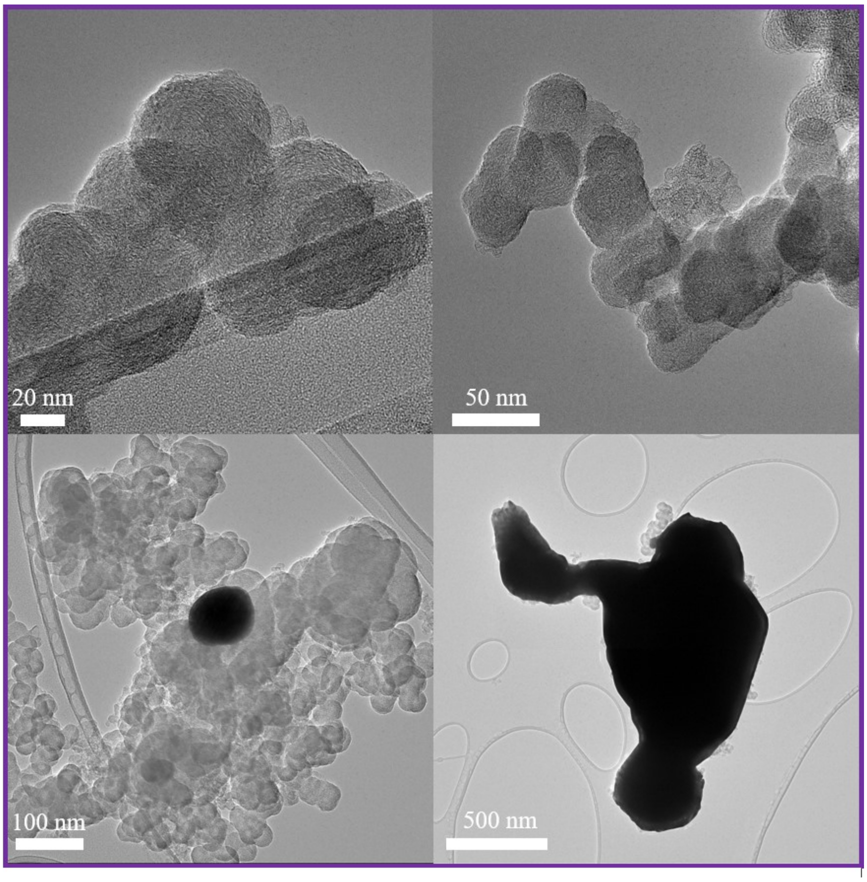

Figure S30: TEM images of commercial 80 % Ag/C_{Vulcan}.

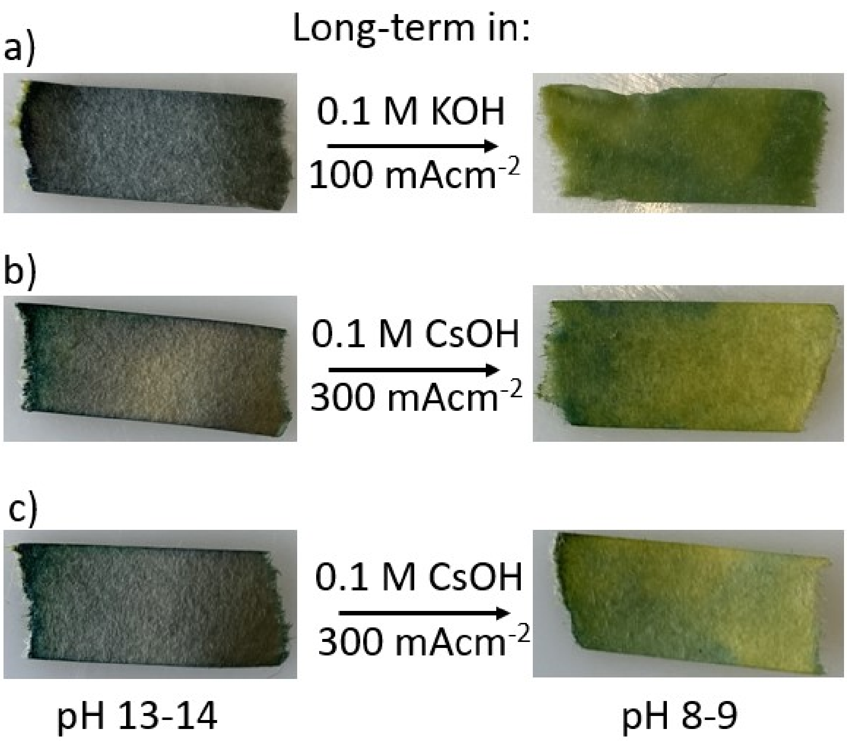

Figure S31: pH change after long-term operation with a) Ag/$C_{Super\ P}$ in 0.1 M KOH b) Ag/$C_{Super\ P}$ in 0.1 M CsOH and c) Ag/C_{Ketjen} in 0.1 M CsOH. The pH of the electrolyte shifts from ~13-14 to ~8-9.

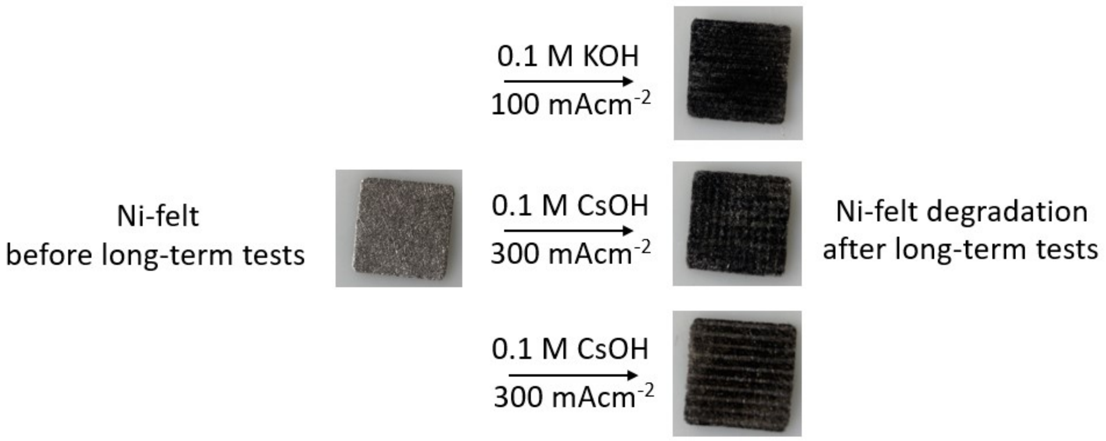

Figure S32: Ni-felt degradation after long-term experiments.

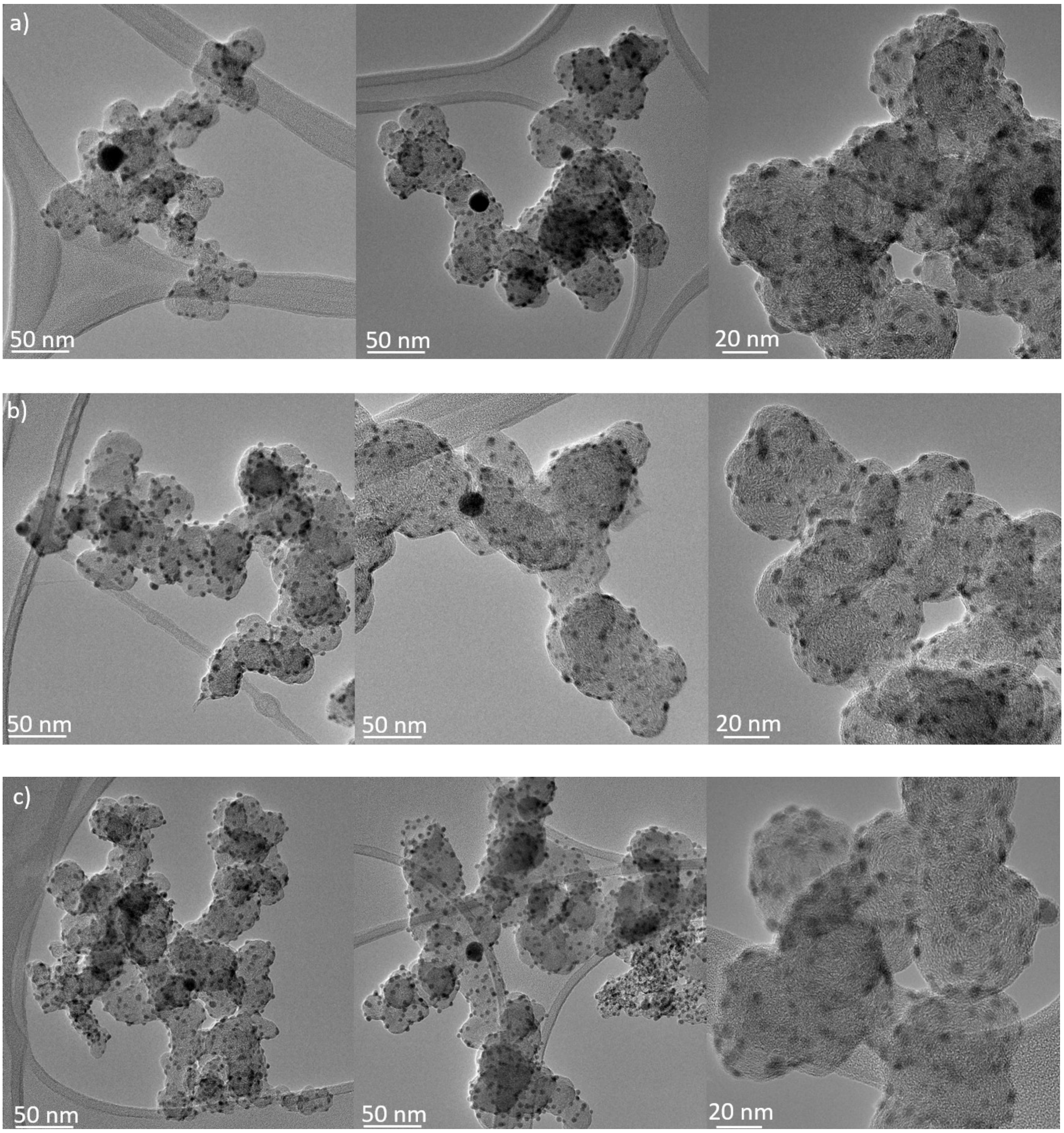

Figure S33: Post-mortem TEM images after long-term operation of a) Ag/$C_{Super\ P}$ tested in 0.1 M KOH b) Ag/$C_{Super\ P}$ tested in 0.1 M CsOH and c) Ag/C_{Ketjen} tested in 0.1 M CsOH.

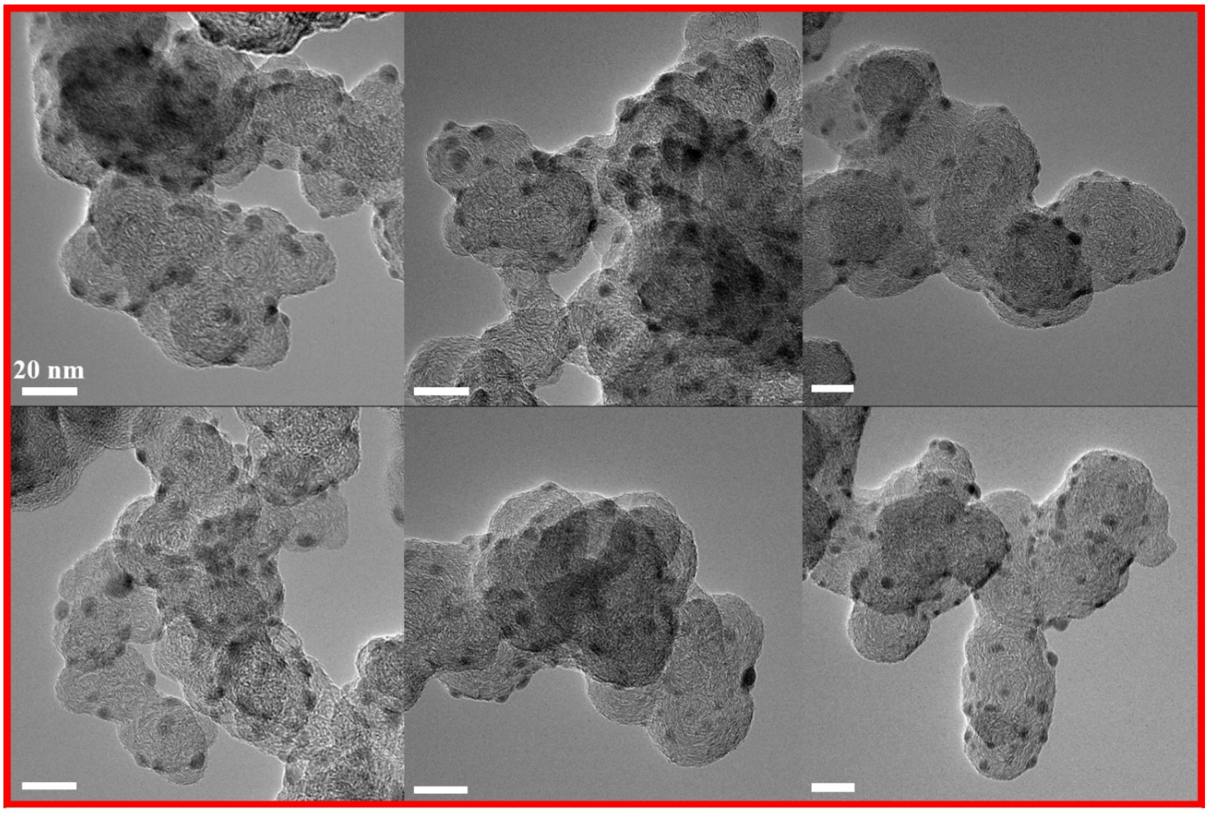

Figure S34: Additional TEM images of Ag/$C_{Super\ P}$.

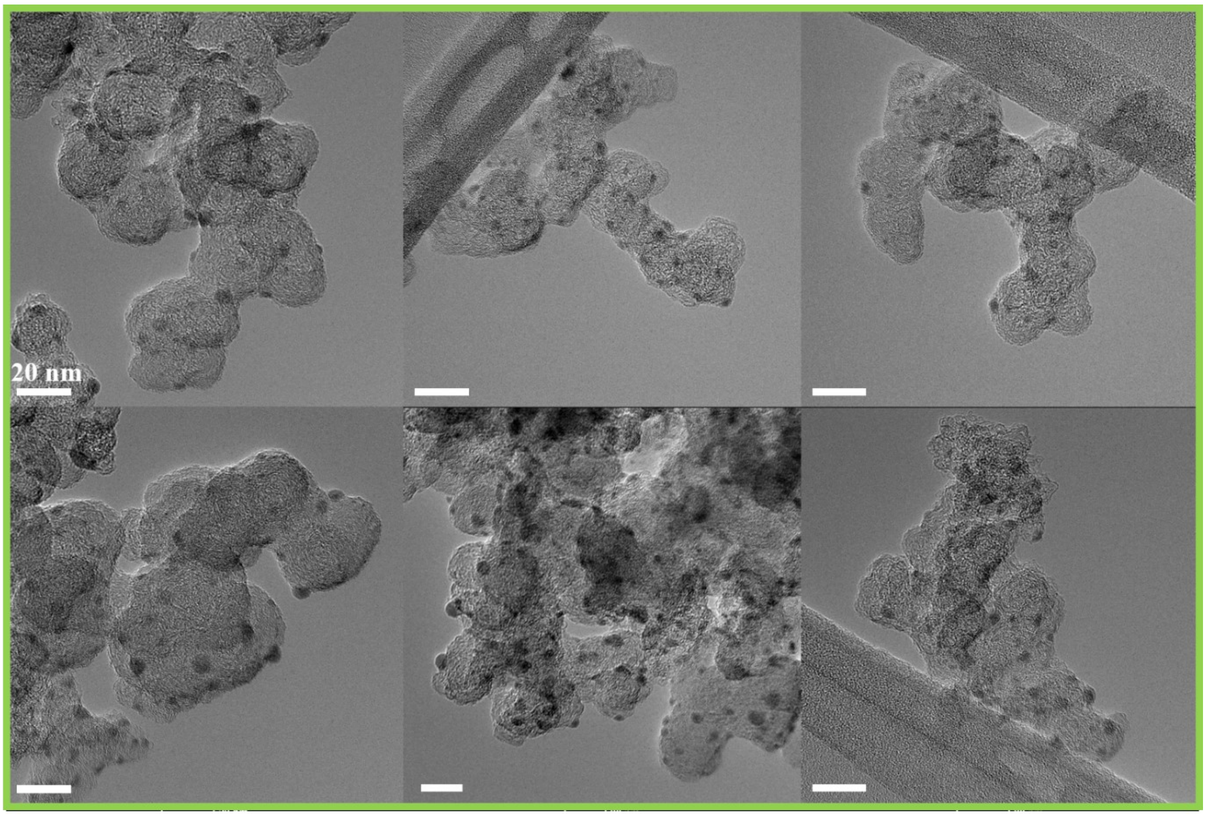

Figure S35: Additional TEM images of Ag/C_{Vulcan}.

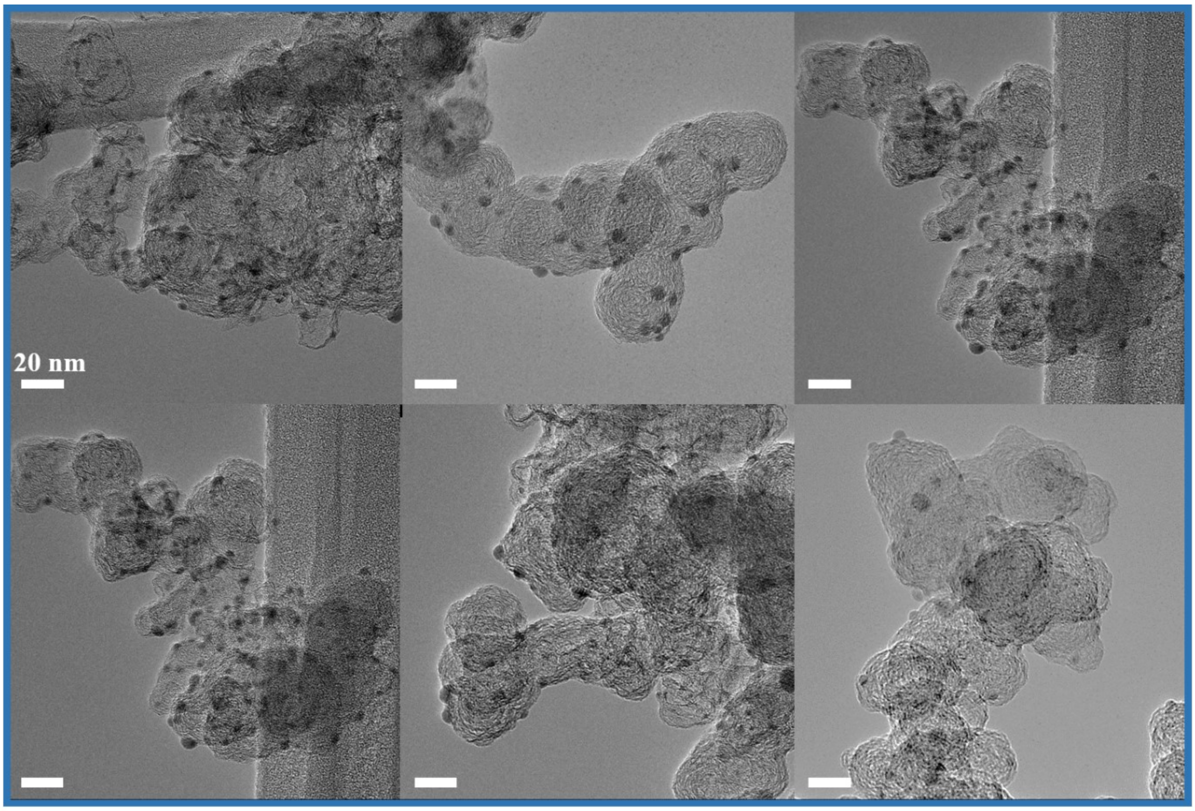

Figure S36: Additional TEM images of Ag/C_{Ketjen}.

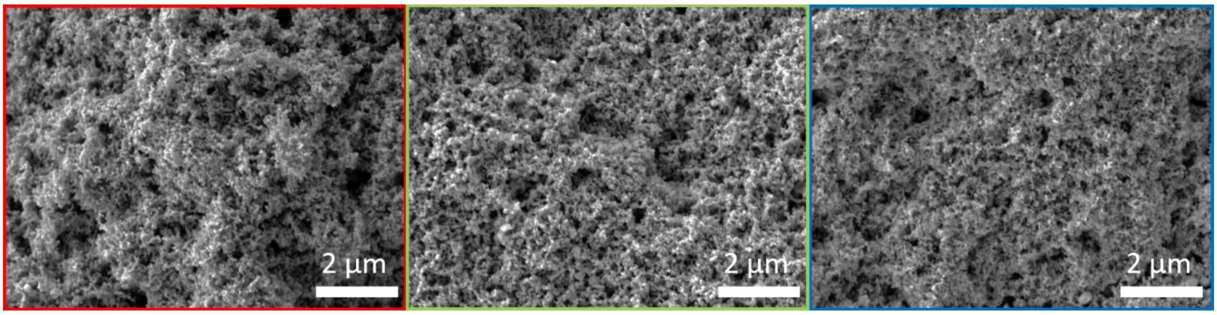

Figure S37: SEM Top view images. Ag on Super P (red), Ag on Vulcan (green), Ag on Ketjenblack (blue).

Literature

[1] C. Kim, H. S. Jeon, T. Eom, M. S. Jee, H. Kim, C. M. Friend, B. K. Min, Y. J. Hwang, *Journal of the American Chemical Society* **2015**, *137*, 13844.

[2] R. Chen, N. T. Nuhfer, L. Moussa, H. R. Morris, P. M. Whitmore, *Nanotechnology* **2008**, *19*, 455604.

[3] J. Zemann, *Acta Cryst* **1965**, *18*, 139.

[4] A. Zana, J. Speder, N. E.A. Reeler, T. Vosch, M. Arenz, *Electrochimica Acta* **2013**, *114*, 455.

[5] Y. Tzeng, C.-Y. Jhan, Y.-C. Wu, G.-Y. Chen, K.-M. Chiu, S. Y.-E. Guu, *Nanomaterials (Basel, Switzerland)* **2022**, *12*.

[6] T. Li, K. Wang, J. Wang, Y. Liu, Y. Han, J. Song, H. Hu, G. Lin, Y. Liu, *J Mater Sci* **2020**, *55*, 4558.
